# Haplotype-Based, Genome-Wide Association Study Reveals Stable Genomic Regions for Grain Yield in CIMMYT Spring Bread Wheat

**DOI:** 10.3389/fgene.2020.589490

**Published:** 2020-12-03

**Authors:** Deepmala Sehgal, Suchismita Mondal, Leonardo Crespo-Herrera, Govindan Velu, Philomin Juliana, Julio Huerta-Espino, Sandesh Shrestha, Jesse Poland, Ravi Singh, Susanne Dreisigacker

**Affiliations:** ^1^International Maize and Wheat Improvement Center (CIMMYT), Texcoco, Mexico; ^2^Campo Experimental Valle de México, INIFAP, México, Mexico; ^3^Kansas State University, Manhattan, KS, United States

**Keywords:** haplotype blocks, haplotype-based GWAS, GBS, EYT, heat map

## Abstract

We untangled key regions of the genetic architecture of grain yield (GY) in CIMMYT spring bread wheat by conducting a haplotype-based, genome-wide association study (GWAS), together with an investigation of epistatic interactions using seven large sets of elite yield trials (EYTs) consisting of a total of 6,461 advanced breeding lines. These lines were phenotyped under irrigated and stress environments in seven growing seasons (2011–2018) and genotyped with genotyping-by-sequencing markers. Genome-wide 519 haplotype blocks were constructed, using a linkage disequilibrium-based approach covering 14,036 Mb in the wheat genome. Haplotype-based GWAS identified 7, 4, 10, and 15 stable (significant in three or more EYTs) associations in irrigated (I), mild drought (MD), severe drought (SD), and heat stress (HS) testing environments, respectively. Considering all EYTs and the four testing environments together, 30 stable associations were deciphered with seven hotspots identified on chromosomes 1A, 1B, 2B, 4A, 5B, 6B, and 7B, where multiple haplotype blocks were associated with GY. Epistatic interactions contributed significantly to the genetic architecture of GY, explaining variation of 3.5–21.1%, 3.7–14.7%, 3.5–20.6%, and 4.4– 23.1% in I, MD, SD, and HS environments, respectively. Our results revealed the intricate genetic architecture of GY, controlled by both main and epistatic effects. The importance of these results for practical applications in the CIMMYT breeding program is discussed.

## Introduction

Bread wheat (*Triticum aestivum* L., 2n = 6x = 42, AABBDD), with global production of 761.5 million tons, is a staple food source for over 2.5 billion people worldwide and an important crop for food security (FAO, 2020). Climate change and population growth will make attainment of food security a challenging task over the coming decades. Development of high-yielding, climate-resilient wheat varieties has therefore become imperative for wheat breeders. Improvement of grain yield (GY) is an arduous task for the global plant-breeding community due to low heritability and intractable “genotype × environment” interactions associated with it, particularly under stress environments (Quarrie et al., 2006; Sehgal et al., 2017, 2020). Nevertheless, wheat breeders have revealed genetic gains up to 1% for GY annually, but further efforts are required to cope with an estimated 2% yearly increase in world population (Tadesse et al., 2019).

Advances in next-generation sequencing technologies have revolutionized the field of plant genomics. Low-cost genotyping platforms that generate thousands to millions of data points are now available for all agronomically important crops, providing effective means for crop genetic research studies (Ganal et al., 2012). For wheat, where marker number and density were major lacunae in conducting in-depth genetic analyses, the availability of dense sets of single-nucleotide polymorphisms (SNPs) from different genotyping platforms has made a powerful step change in the marker tool kit (Poland et al., 2012; Cavanagh et al., 2013; Wang et al., 2014). The resulting high-density genomic data have opened up new possibilities for untangling the genetic architecture of complex traits by genome-wide association study (GWAS) and to perform other genomic studies, for instance, the analysis of selective sweeps within or across species (Afzal et al., 2019; Liu et al., 2019). Additionally, the recent availability of the high-quality reference genome of bread wheat (IWGSC, 2018) has enhanced our understanding of the regulation of genome organization, gene expression, and evolutionary mechanisms shaping its genome (Alaux et al., 2018; Ramírez-González et al., 2018; Wicker et al., 2018). With genome resolution reaching megabase-scale level in wheat, it is envisioned that genomics-assisted breeding can be escalated to a scale that was not possible previously (Keeble-Gagnère et al., 2018).

Although high-density markers, such as genotyping-by-sequencing (GBS) or SNP arrays, have been used extensively in wheat to explore the genetic architecture of GY and yield components using GWAS (Neumann et al., 2011; Zhang et al., 2013; Edae et al., 2014; Ain et al., 2015; Azadi et al., 2015; Lopes et al., 2015; Sukumaran et al., 2015; Sehgal et al., 2016; Qaseem et al., 2018; Garcia et al., 2019; Li et al., 2019, 2020; Ward et al., 2019; Shokat et al., 2020), panel sizes have been relatively small to dissect such a complex trait, and results therefore were quite variable, identifying hundreds of small-effect QTL. GWAS reports in larger germplasm panels are still rare (Sehgal et al., 2017, 2020; Juliana et al., 2019). Small panel sizes have also hindered scientists from exploring epistatic interactions due to lack of reasonable statistical power (Mackay, 2014).

To boost the power of single-marker GWAS, meta-GWAS has emerged as a leading approach to dissect traits (Evangelou and Ioannidis, 2013). In this approach, summary statistics of multiple trials are analyzed in a single frame to determine the most effective stable loci over space and time while simultaneously reducing false positives. In wheat, this approach has been used successfully to identify important loci associated with quality traits in unbalanced datasets (Battenfield et al., 2018). However, this GWAS approach fails to address the issue of “missing heritability,” which is common in single marker–based GWAS. The alternative approach to boost the power of GWAS is by constructing haplotypes between neighboring SNPs on a chromosome. As specific sets of alleles observed on a single chromosome, haplotypes are inherited together with little chance of contemporary recombination. Recent studies on wheat and other crops have shown that GWAS analysis with haplotypes can be superior to single marker–based analysis in terms of statistical significance (better *p*-values) and in estimating allelic effects (Hao et al., 2012; Lu et al., 2012; N’Diaye et al., 2017; Ledesma-Ramírez et al., 2019; Li et al., 2019; Sehgal et al., 2020; Shokat et al., 2020).

In the present study, we targeted exploration of stable regions in the genome that define the backbone of the genetic architecture of GY in CIMMYT spring bread wheat germplasm using a haplotype-based GWAS and investigating the interactions among haplotypes. We used seven large cohorts of advanced breeding lines from different breeding cycles phenotyped under well-managed multiple testing environments (irrigated and stress conditions) and genotyped with GBS markers. The specific objectives were to (i) construct haplotypes using GBS data across 6,461 lines distributed in seven elite yield trials (EYTs); (ii) conduct haplotype-based GWAS in each EYT using phenotyping data derived from the four testing environments; (iii) identify stable haplotypes associated with GY under individual testing environments and across multiple testing environments; and (iv) investigate the contribution of epistatic interactions to the genetic architecture of GY.

## Materials and Methods

### Plant Materials, Phenotyping, and Statistical Analysis

A total of 6,461 spring bread wheat lines, which formed the entries of seven EYTs during 7 consecutive years, were used in this study (Supplementary Table 1). EYT2011-12, EYT2012-13, EYT2013-14, EYT2014-15, EYT2015-16, EYT2016-17, and EYT2017-18 consisted of 643, 998, 983, 942, 829, 1,086, and 980 non-overlapping lines, respectively. Each trial year the breeding program selects 1,092 advanced lines for second-year yield testing, which is the source for the lines above. The 1,092 lines in each year were divided into 39 experiments, each with 28 entries and 2 checks in an alpha lattice design with 3 replications. All EYT were phenotyped at the Norman E. Borlaug Experimental Research Station (CENEB) in Ciudad Obregon, Mexico, under multiple contrasting environments by modulating planting dates and irrigation. All trials were sown in bed planting. The plot size was 2.8 m × 1.6 m (2 beds of 0.8 m with 3 rows each).

The multiple environments included optimum irrigated (I) and three stress environments [mild drought stress (MD), severe drought (SD) stress, and heat stress (HS)]. In environment I, five irrigations were applied (at germination and 40, 70, 95, and 115 days after the first irrigation) with a total water supply of maximum 500 mm distributed through five irrigation events across the crop cycle, while in MD environment two irrigations were applied, one at germination and the other after 50 days (using furrow irrigation; the total water supply was 280 mm). In SD environment, drip irrigation was applied at germination and after 50 days with a total water supply of 180 mm available for the plant. In HS environment, planting was delayed by 3 months (end of February) and around 500 mm of water was applied across the crop cycle through five to six irrigation events. Ciudad Obregon station has little to no rainfall during the crop growing season (November to April). It has a desert-type climate with rains concentrated during the months of August to October (Mondal et al., 2020). However, if there is rain, the irrigation in stressed environments is adjusted to maintain the amounts. Trials were phenotyped for GY, days to heading (DH), and plant height (PH) in each year, as detailed in Sehgal et al. (2020).

The phenotypic data of GY collected for each genotype were adjusted for block effects within each of three replications per trial (incomplete blocks considered as random effects) using the PROC MIXED function in SAS 9. For DH and PH, the adjusted means were calculated by the formula Y = (Y_ij_ - Y_i_) + Y_all trials_, where Y_ij_ is the value of the entry for a trial, Y_i_ is the mean of checks of that trial, and Y_all trials_ is the mean of checks of all trials. The summary statistics function in GenStat 14th ed. was used to obtain the minimum and maximum values of each trait in each trial. ANOVA was performed using a customized script in R version 3.4 (Supplementary Datasheet 1).

### Genomic DNA Extraction and Genotyping

Genomic DNA was extracted from dried leaves collected from five plants per line using a modified CTAB method described in CIMMYT laboratory protocols (Dreisigacker et al., 2016). All lines were genotyped using GBS Kansas State University using 192-plexing on Illumina HiSeq2000. SNP calling was done using TASSEL 5 pipeline as described in Rutkoski et al. (2016). To obtain physical positions of SNPs, sequence reads of the SNPs were blasted to the wheat reference genome RefSeq V.1.1 (IWGSC, 2018).

### Population Structure, Linkage Disequilibrium (LD), and Haplotype Blocks

The population structure was assessed through principal component analysis (PCA) using the rgl package in R (Adler and Murdoch, 2013). GAPIT version 2.0 was used to obtain correlation estimates of the frequency of the squared allele of LD (*r*^2^) for all pairwise comparisons. LD decay was visualized by plotting pairwise *r*^2^ values against the physical distance (Mb) for the whole genome, separately for each EYT, and using combined data from the 6,333 lines. A smooth line was fit to the data using second-degree, locally weighted scatterplot smoothing (Breseghello and Sorrells, 2006). For the LOESS estimation of LD decay, genetic distance was estimated as the point where the LOESS curve first crosses the baseline *r*^2^ of 0.1.

To avoid obtaining different haplotype blocks in each of the seven EYTs due to different minor allele frequencies (MAF) of the markers, the GBS data of all seven EYTs were considered together to generate the haplotype blocks. The MAF threshold was set to 0.15 instead of the usual 0.05 so that a 0.05 MAF could be achieved in each EYT. The haplotype blocks were constructed in R, based on the confidence interval algorithm developed by Gabriel et al. (2002) and detailed in Sehgal et al. (2019, 2020). Briefly, D’ 95% confidence intervals between SNPs was calculated, and comparisons were divided into categories of “strong LD,” “inconclusive,” or “strong recombination.” If 95% of the comparisons in one block were in “strong LD,” a haplotype block was created. The minimum lower and upper confidence interval values were set to 0.6 and 0.95, respectively. The constructed blocks transformed into multiallelic markers, considering the allelic combinations within each block to be independent alleles.

### Haplotype-Based GWAS

GWAS was performed in each individual EYT using a mixed linear model (MLM) using Plink version 1.07 (Purcell et al., 2007) with PCA as fixed variate and kinship as random. PCA was conducted using the rgl package in R, and the appropriate number of principal components to be used in MLM was assessed based on Bayesian information criteria (Schwarz, 1978). The kinship matrix was calculated by the VanRaden algorithm (VanRaden, 2008).

A haplotype was considered stable for a testing environment when it showed *P* value < 10^–4^ in one EYT and at least *P* < 10^–3^ across three or more EYTs (Sehgal et al., 2020). Similarly, a haplotype was categorized as stable for multiple testing environments when it showed significance of at least *P* < 10^–3^ in two or more testing environments across two or more EYTs. The allelic effect of the associated haplotypes was estimated as the difference between the mean value of lines with and without favorable allele and was presented as box plots.

### Epistatic Interactions

A linear regression model was used to calculate *P* values and percentage variation as R^2^ for two- and three-locus haplotype interactions using an in-house designed script in R (Supplementary Datasheet 1). A significant threshold of *P* < 0.0001 was used to declare significant interactions.

## Results

### Phenotypic Trait Variation in EYT Under Contrasting Environments

Phenotypic traits revealed a wide distribution in all EYTs in all environments (Supplementary Figures 1a,b). GY showed significant (*P* < 0.001) and positive correlations with PH in 26 EYTs × environment combinations, while the correlations with DH were positive in irrigated environments and negative in stress environments (MD, SD, and HS) across years.

**FIGURE 1:**
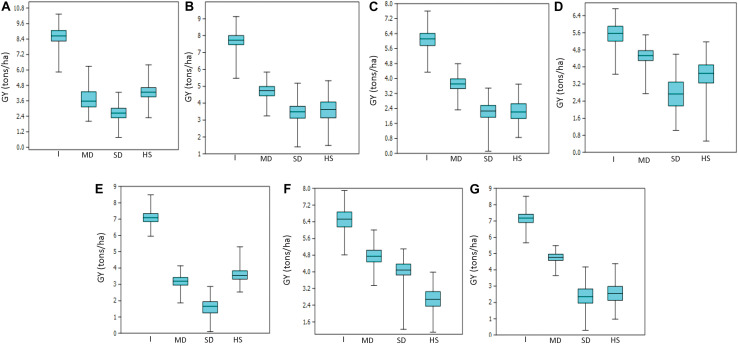
Grain yield variation under different environments in EYT2011-12 **(A)**, EYT2012-13 **(B)**, EYT2013-14 **(C)**, EYT2014-15 **(D)**, EYT2015-16 **(E)**, EYT2016-17 **(F),** and EYT2017-18 **(G)**.

Mean GY across all trials and environments ranged from 1622 kg/ha (EYT2015-16 in SD) to 8622 kg/ha (EYT2011-12 in B-5IR) (Supplementary Table 2). In general, SD and HS were the least yielding environments, except in EYT2011-12 and EYT2015-16 (Figure 1). ANOVA showed highly significant effects (*P* < 0.001) of genotypes, environments, and genotype × environment interactions for GY in the seven EYTs (Supplementary Table 2). Broad sense heritability estimates ranged from 0.31 (EYT2015-16) to 0.63 (EYT2011-12) (Supplementary Table 2).

### Haplotype Blocks; Genome-Wide Coverage and Distribution

An initial set of 50,058 SNP markers was obtained on 6,461 lines. Of these, a filtered set of 14,027 SNP with maximum 30% missing data and a minor allele frequency (MAF) ≥ 0.15 was extracted without imputation. Lines showing more than 60% missing data were also culled with 6,333 genotypes remaining for further analysis.

A total of 519 haplotype blocks were established across the genomes. The haplotype blocks covered a total genome length of 14,036 Mb with 4,925, 5,170, and 3,941 Mb covered in the A, B, and D genomes, respectively (Table 1). The blocks were distributed according to the length of each chromosome, and the density of the markers with the highest numbers were obtained in A and B genomes (231 and 239, respectively) and the lowest in the D genome (49). The highest number was obtained on chromosomes 7A (51), followed by chromosomes 2B and 7B (46 each), whereas the lowest number of haplotype blocks was obtained on chromosome 4D (1).

**TABLE 1:** Summary of haplotype blocks (HB) in 6,333 lines of seven elite yield trials.

**Chromosome**	**No. of SNPs**	Physical position (bp)- First and Last SNP across chromosome*	**Number of HB**	**Total number of alleles**	**Number of SNPs in an HB (min–max)**
1A	585	114,5442–593,790,090	32	85	2–5
1B	804	143,0915–688,983,196	23	60	2–5
1D	444	78,777–492,641,722	8	21	2–5
2A	784	138,1013–779,674,593	29	82	2–6
2B	1,042	19,097–800,780,364	46	128	2–8
2D	550	152,5890–651,419,179	13	34	2–6
3A	731	711,587–750,500,626	31	86	2–7
3B	903	198,860–829,535,127	33	92	2–6
3D	576	344,069–615,458,508	6	16	2–6
4A	639	881,423–744,304,645	29	79	2–5
4B	423	622,455–672,953,537	12	30	2–6
4D	171	739,257–509,849,696	1	3	4
5A	669	217,5212–709,755,448	29	72	2–6
5B	793	16,637–712,600,907	41	103	2–6
5D	337	93,2817–565,975,773	5	13	2–4
6A	609	684,328–617,838,620	30	81	2–5
6B	880	195,536–720,519,123	38	100	2–6
6D	379	687,775–473,287,249	10	25	2–6
7A	1,055	289,461–736,572,283	51	143	2–6
7B	899	259,901–747,616,899	46	131	2–6
7D	754	114,2643–638,541,382	6	16	2–5

*Physical positions of the first and the last SNP on individual chromosomes.

### Population Structure, Linkage Disequilibrium Decay, and Significant Associations

All seven EYTs showed a moderate structure with two to three subgroups deciphered by PCA (Supplementary Figure 2). Whole genome linkage disequilibrium (LD) decay in the individual EYT and combined EYTs is shown in Supplementary Figure 3, which revealed that LD decay varied from 1.8 Mb in EYT2014-15 to 2.3 Mb in EYT2016-17, with an average LD decay of approximately 2 Mb.

**FIGURE 2:**
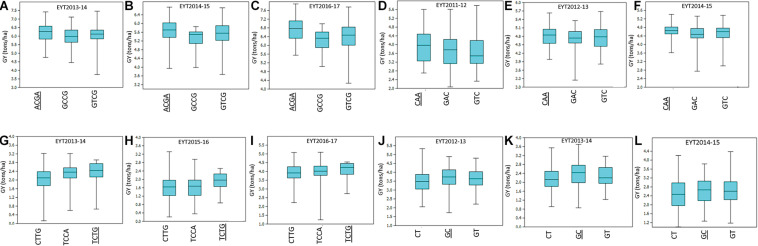
Allelic effects of haplotype blocks associated with GY under I **(A–C)**, MD **(D–F)**, SD **(G–I)**, and HS **(J–L)** environments. The favorable haplotype in each part of the figure is shown as underscored. The parts **(A–L)** show allelic effects of HB3A.1, HB2A.29, HB 4D.1, and HB7A.2, respectively.

For environment I, seven stable associations were identified across EYTs (Supplementary Table 3) on chromosomes 2A (1), 3A (1), 4A (1), 4B (1), 5B (1), and 6B (2). Of these, favorable alleles of two associated blocks on chromosomes 3A and 4B showed GY advantage of >100 kg/ha across EYTs. For haplotype block HB3A.1, the favorable haplotype ACGA resulted in GY increase of 215 to 525 kg/ha in three EYTs (Figure 2). Similarly, the favorable haplotype TC in haplotype block HB4B.12 resulted in an increase of 168 to 429 kg/ha in GY across EYTs. The HB5B.29 linked to flowering time gene *Vrn-B1* had two favorable alleles (AC and GT) and showed allelic effects of 47 to 568 kg/ha across EYTs (Supplementary Table 3).

In environment MD, four haplotype blocks on chromosomes 1A (1), 1B (1), 2A (1), and 3B (1) showed association with GY. Of these, HB1A.13 showed GY advantage of >100 kg/ha across, while HB1B.19 showed effects up to 553 kg/ha in EYT2011-12 (Supplementary Table 3). In the SD environment, 10 blocks showed association with GY. These were identified on chromosomes 1A (1), 1B (1), 4A (2), 4D (1), 5B (2), 6A (1), and 7B (2). Of these, three associations on chromosomes 1B, 4D, and 6A showed the largest allele effects. For HB1B.3, two favorable alleles (GG and AG) resulted in a 126–359 kg/ha increase in GY across EYTs, while favorable alleles at HB4D.1 (TCTG) and HB6A.6 (GA) resulted in an increase of 151–362 kg/ha and 203–248 kg/ha, respectively (Figure 2). The block HB5B.29 linked to the major vernalization gene *Vrn-B1* showed association with GY across five EYTs, with the favorable allele resulting in an increase of 263–430 kg/ha (Supplementary Table 3). Since both I and SD environments showed significant association with this block, both phenological traits (DH and PH) were used as covariates in GWAS to test the significance of this locus. However, when DH and PH were used as covariates, HB5B.29 locus either disappeared or became less significant.

For HS, 15 haplotype blocks were associated on chromosomes 2B (1), 3A (1), 3B (2), 4A (1), 5B (1), 6A (1), 6B (2), 7A (5), and 7B (1). The associations on chromosomes 2B (HB2B.12) and 4A (HB4A.24) and all associations on chromosome 7A (HB7A.2, HB7A.3, HB7A.20, HB7A.28, and HB7A.32) showed large allelic effects compared to other blocks (Supplementary Table 3).

**FIGURE 3:**
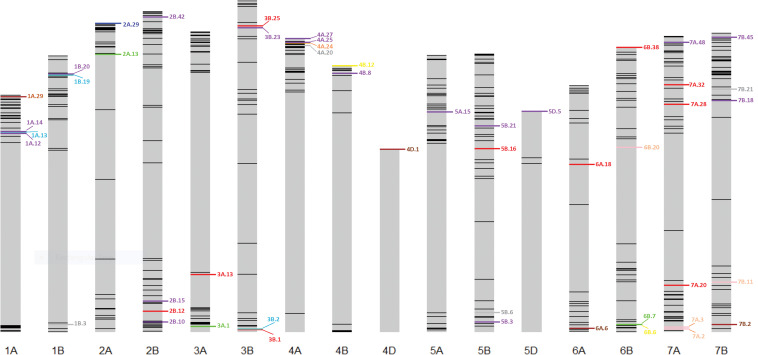
Environment-specific stable haplotypes and haplotypes identified to be significant across multiple environments and EYTs. Green, blue, brown, and red colors show environment-specific associations with GY under I, MD, SD, and HS environments, respectively. Purple color shows stable (S) associations significant across multiple environments and EYTS, while turquoise, pink, yellow, orange, and gray show associations that were identified under two categories; turquoise (MD, S), pink (HS, S), yellow (I, S), orange (SD, HS), and gray (SD, S).

**FIGURE 4:**
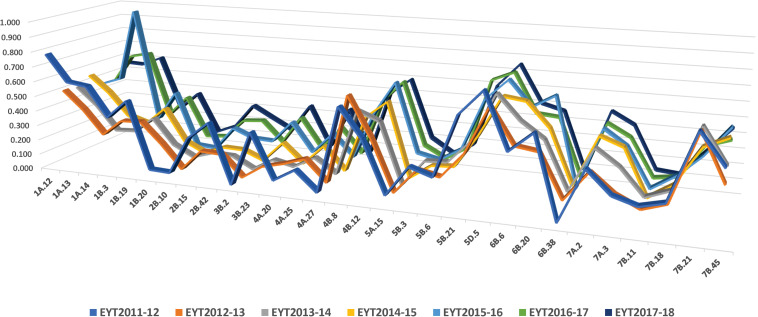
Frequency of stable haplotypes over 7 years (EYT2011-12 to EYT2017-18).

Thirty stable haplotypes that are favorable in multiple environments and across EYTs were identified (Table 2 and Supplementary Table 4), including seven hotspots on chromosomes 1A, 1B, 2B, 4A, 5B, 6B, and 7B, where multiple haplotype blocks on same chromosome were associated with GY. The associations on chromosomes 2B (HB2B.10), 3B (HB3B.2), 4B (HB4B.12), 5D (HB5D.5), and 7B (HB7B.18) resulted in a GY increase of 177 to 357, 148 to 449, 168 to 429, 116 to 496, and 122 to 470 kg/ha in different environments and EYT, respectively (Supplementary Table 4). Figure 3 shows all stable haplotypes on chromosome maps, and Figure 4 shows the frequencies of the favorable haplotypes of each of the 30 associated blocks in all seven EYTs. The frequencies of 23 haplotypes varied from 11 to up to 78% across EYTs, while the frequency of seven haplotypes (HB1B.20, HB2B.10, HB3B.2, HB4A.20, HB4A.27, HB5A.15, and HB6B.38) remained low across EYTs (Supplementary Table 5). Potential candidate genes underlying 28 out of 30 haplotype blocks were identified and are listed in Supplementary Table 6.

**TABLE 2:** Thirty stable associations identified for grain yield considering all elite yield trials and testing environments (I: Irrigated; MD: Moderate drought; SD: Severe Drought; HS: Heat Stress) together.

**Haplotype block**	**Markers in blocks**	**Chromosome**	**Interval; First-Last SNP (bp)**	**Elite yield trials; Testing environment**
HB1A.12	S1A_494392059, S1A_494393037	1A	978	EYT2011-12; DS, EYT2012-13; HS, EYT2013-14; MD, EYT2013-14; SD, EYT2016-17; MD
HB1A.13	S1A_497201550, S1A_497201682	1A	132	EYT2012-13; MD, EYT2013-14; MD, EYT2013-14; SD, EYT2016-17; MD, EYT2016-17; HS
HB1A.14	S1A_499864157, S1A_499864420, S1A_499864432, S1A_500074551	1A	210394	EYT2011-12; MD, EYT2012-13; HS, EYT2015-16; MD, EYT2016-17; MD, EYT2016-17; HS
HB1B.3	S1B_18569448, S1B_18570787	1B	1339	EYT2011-12; I, EYT2013-14; SD, EYT2014-15; MD, EYT2014-15; HS, EYT2015-16; SD, EYT2017-18; SD
HB1B.19	S1B_639415604, S1B_639415692, S1B_639426265	1B	10661	EYT2011-12; MD, EYT2012-13; HS, EYT2013-14; MD, EYT2014-15; I, EYT2014-15; MD, EYT2014-15; HS, EYT2015-16; MD
HB1B.20	S1B_642616640, S1B_642616658	1B	18	EYT2014-15; SD, EYT2016-17; MD, EYT2016-17; HS, EYT2017-18; SD
HB2B.10	S2B_24883552, S2B_24887574, S2B_24899507, S2B_24899536, S2B_24899572	2B	16020	EYT2012-13; HS, EYT2016-17; I, EYT2017-18; MD, EYT2017-18; HS
HB2B.15	S2B_75832544, S2B_75848057	2B	15513	EYT2012-13; DS, EYT2012-13; HS, EYT2015-16; DS, EYT2016-17; HS
HB2B.42	S2B_784544719, S2B_784774250, S2B_784905791, S2B_784905811	2B	361092	EYT2011-12; MD, EYT2011-12; HS, EYT2012-13; I, EYT2014-15; SD, EYT2015-16; SD
HB3B.2	S3B_7240747, S3B_7240753	3B	6	EYT2011-12; MD, EYT2011-12; HS, EYT2012-13; SD, EYT2017-18; HS
HB3B.23	S3B_758391015, S3B_758438223, S3B_758464620, S3B_758612003, S3B_758612056, S3B_758613386	3B	222371	EYT2011-12; MD, EYT2011-12; HS, EYT2013-14; MD, EYT2016-17; I, EYT2016-17; SD, EYT2017-18; HS
HB4A.20	S4A_713064971, S4A_713506269, S4A_713517340, S4A_713522176	4A	457205	EYT2011-12; SD, EYT2013-14; I, EYT2013-14; SD, EYT2014-15; HS, EYT2017-18; SD
HB4A.25	S4A_721406670, S4A_721406696, S4A_721826603, S4A_721826636	4A	419966	EYT2012-13; MD, EYT2013-14; SD, EYT2015-16; I, EYT2015-16; MD
HB4A.27	S4A_730188545, S4A_730188899	4A	354	EYT2011-12; SD, EYT2012-13; SD, EYT2012-13; HS, EYT2014-15; I, EYT2014-15; MD
HB4B.8	S4B_644330895, S4B_644330917	4B	22	EYT2011-12; I, EYT2012-13; SD, EYT2012-13; HS, EYT2013-14; I, EYT2013-14; MD, EYT2013-14; SD, EYT2016-17; HS
HB4B.12	S4B_663621978, S4B_663622013	4B	35	EYT2013-14; I, EYT2014-15; I, EYT2014-15; SD, EYT2016-17; I
HB5A.15	S5A_548234618, S5A_548234636, S5A_548387200, S5A_548422588	5A	187970	EYT2012-13; HS, EYT2014-15; MD, EYT2016-17; I, EYT2017-18; I
HB5B.3	S5B_24292046, S5B_24537970, S5B_24648800, S5B_24677091	5B	385045	EYT2011-12; HS, EYT2012-13; I, EYT2013-14; HS, EYT2017-18; MD
HB5B.6	S5B_47584429, S5B_47592949	5B	8520	EYT2011-12; HS, EYT2012-13; SD, EYT2012-13; HS, EYT2013-14; SD, EYT2014-15; SD
HB5B.21	S5B_513712393, S5B_513713184	5B	791	EYT2011-12; SD, EYT2012-13; MD, EYT2014-15; I, EYT2015-16; HS
HB5D.5	S5D_550192169, S5D_550192174	5B	5	EYT2012-13; HS, EYT2014-15; I, EYT2016-17; I, EYT2016-17; MD
HB6B.6	S6B_17686703, S6B_17701765	6B	15062	EYT2011-12; I, EYT2014-15; I, EYT2016-17; I, EYT2017-18; I
HB6B.20	S6B_459374225, S6B_459374299	6B	74	EYT2011-12; HS, EYT2012-13; SD, EYT2012-13; HS, EYT2017-18; HS
HB6B.38	S6B_708712113, S6B_708712131	6B	18	EYT2011-12; SD, EYT2011-12; HS, EYT2012-13; HS, EYT2014-15; HS
HB7A.2	S7A_7938818, S7A_7938819	7A	1	EYT2012-13; HS, EYT2013-14; HS, EYT2014-15; HS, EYT2017-18; SD, EYT2017-18; HS
HB7A.3	S7A_12011058, S7A_12011069	7A	11	EYT2011-12; SD, EYT2011-12; HS, EYT2012-13; HS, EYT2013-14; HS, EYT2015-16; HS, EYT2017-18; SD, EYT2017-18; HS
HB7B.11	S7B_124548883, S7B_124549059	7B	176	EYT2011-12; SD, EYT2011-12; HS, EYT2012-13; HS, EYT2013-14; SD, EYT2014-15: HS, EYT2017-18; HS
HB7B.18	S7B_576927863, S7B_576927877	7B	14	EYT2012-13; HS, EYT2014-15; SD, EYT2015-16; HS, EYT2016-17; MD
HB7B.21	S7B_605313385, S7B_605313397	7B	12	EYT2011-12; SD, EYT2012-13; SD, EYT2013-14; HS, EYT2016-17; HS, EYT2017-18; SD
HB7B.45	S7B_733461150, S7B_733461162	7B	12	EYT2011-12; SD, EYT2012-13; HS, EYT2013-14; I, EYT2014-15; MD, EYT2016-17; I

**FIGURE 5:**
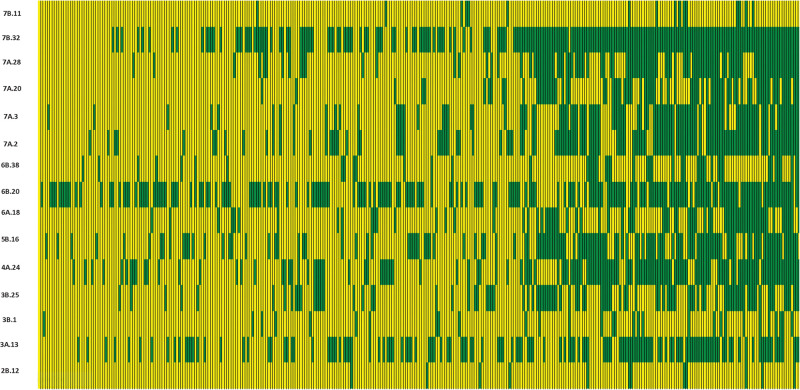
Heat map showing contrasting lines from EYT2015-16 showing all 15 haplotype blocks identified in HS environment. Each yellow vertical line represents a genotype, and each green vertical green rectangle represents a favorable haplotype of an associated block from a chromosome. The name of the haplotype block is shown on the left.

**FIGURE 6:**
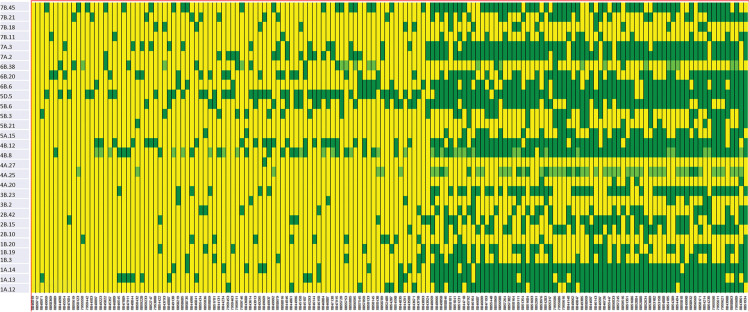
Heat map showing contrasting lines from EYT2015-16 showing favorable alleles of all 30 haplotype blocks identified to be stable across environments and EYTs. Each yellow vertical line represents a genotype. Each dark green rectangle represents the first favorable haplotype of an associated block from a chromosome while light green color represents the second favorable haplotypes identified in a few haplotype blocks. The name of the haplotype block is shown on the left.

**FIGURE 7:**
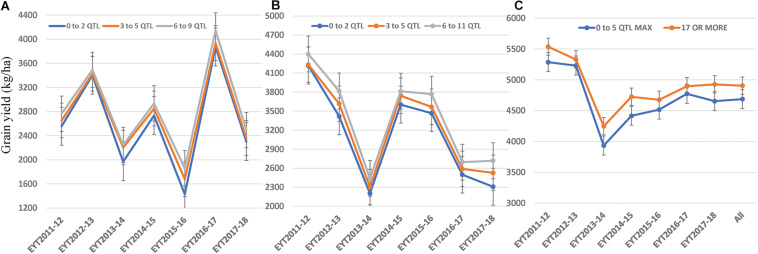
Average grain yield per trial observed by simulating numbers of favorable haplotypes identified in SD **(A)** and HS **(B)** environments and all environments **(C)**.

We constructed heat maps for all seven EYTs to visualize the series of favorable haplotypes accumulated in individual genotypes. Figures 5, 6 show heat maps of selected lines in EYT2015-16 displaying having none to maximum number of favorable haplotypes under HS environment and across all environments and EYTs, respectively (15 and 30 haplotype blocks). Heat maps shown here revealed that the maximum number of favorable haplotypes accumulated in lines from EYT2015-16 were 11 and 23 from the total 15 and 30 haplotypes identified under HS environment, and across environments and EYTs, respectively. We further estimated the additive effects of the favorable haplotypes on GY for (a) the environment-specific haplotypes and (b) all 30 stable multi-environmental haplotypes. The trend showed that with an increasing number of haplotypes, GY increases in all EYTs in all environments. Figure 7 shows the additive effects with an increasing number of haplotypes on GY in the two stress environments, SD and HS (Figures 7A,B) and across all environments (Figure 7C). The increase in GY ranged from 2.5 to 14.1% and 4.3 to 17.7% across EYTs in SD and HS environments, respectively (Figures 7A,B). When stable associations from all environments were tested, GY increase was on average 8% (Figure 7C).

### Epistatic Interactions

Except in MD environment, epistatic interactions were observed in all environments among associated loci (Supplementary Figures 4–6). Most importantly, in both I and SD environments, *Vrn-B1*-linked locus HB5B.29 contributed significantly to epistatic interactions. In environment I, HB5B.29 interacted with HB4B.12 and HB6B.6 more frequently than others, while in environment SD, interactions between HB5B.29 and HB6A.6 were frequent. In environment HS, four associated haplotype blocks from chromosome 7A (HB7A.2, HB7A.3, HB7A.28, and HB7A.32) were mainly involved in interactions among themselves and with other loci. The percent variation explained ranged from 1.5 to 7.5%, 3.6 to 12.9%, and 3.4 to 10.9% in I, SD, and HS environments, respectively.

Genome-wide epistatic interactions were observed in all four environments (Supplementary Figures 7–10). In environments SD and HS, interactions were observed in all EYTs. The percent variation explained ranged from 3.5 to 21.1%, 3.7 to 14.7%, 3.5 to 20.6%, and 4.4 to 23.1% in I, MD, SD, and HS environments, respectively.

## Discussion

Although much research exploring the genetic architecture of yield and yield-associated traits has been reported in wheat using GWAS, the identification of more stable key determinants of GY remain relatively unexplored, largely due to the complexity of the trait and small panel sizes used in previous studies leading to the so-called “large p small n” or “short-fat data” problem (Diao and Vidyashankar, 2013). Additionally, use of bi-allelic SNPs accentuated “missing heritability” issues and therefore reported markers had limited impact in breeding. In the present study, we performed haplotype-based GWAS using 519 haplotype blocks on seven large cohorts of advanced CIMMYT spring bread wheat lines consisting of 6,333 genotypes overall. In addition, epistatic interactions among the genome-wide haplotypes were investigated, an important aspect that has not yet been fully explored in wheat GWAS in order to address the missing heritability (Zuk et al., 2014; Sehgal and Dreisigacker, 2019).

Three approaches are generally used to construct haplotype blocks: (1) user-defined length, (2) sliding-window, and (3) LD. The user-defined fixed length of haplotype blocks (2–15 bp) is the easiest approach; however, generated haplotypes do not reflect genetic principles such as recombination or LD (Gabriel et al., 2002) or a shared evolutionary history (Templeton et al., 2005). The sliding-window approach is the most widely used for building haplotypes in GWAS (Braz et al., 2019). This approach is easy to use and handle; however, when adjacent SNPs are in strong LD, it provides redundant information, making it no more informative than SNPs. Similarly, when LD patterns vary over large genomic regions, it is difficult to determine the appropriate window size for a genome-wide scan. The LD-based approach is the most advantageous because it focuses directly on the detection of historical recombination in the test population (Qian et al., 2017).

We constructed haplotypes using an LD-based approach and conducted a haplotype-based GWAS and epistatic scan to dissect the genetic architecture of GY under contrasting sets of environments and across seven EYTs. The total number of genome-wide haplotype blocks obtained was in a similar range as reported in the recent studies using same marker platform (Singh et al., 2018; Ledesma-Ramírez et al., 2019; Shokat et al., 2020). Li et al. (2019) used a much higher density of markers from two platforms (wheat 90K and 660K Illumina SNP arrays) and thus were able to obtain much higher numbers of haplotype blocks per chromosome and across the genome. However, panel size remained small (166 lines) in their study. The average LD decay in the seven EYTs in the present study was observed at ∼2 Mb. Comparison of LD decay with previous studies in wheat in which physical distance was used for estimating LD decay (Liu et al., 2017; Ladejobi et al., 2019; Li et al., 2019) revealed a faster decay in the CIMMYT germplasm (2 Mb in CIMMYT germplasm vs. 4–8 Mb in the above-mentioned studies). This suggests high levels of genetic diversity in the current CIMMYT breeding germplasm, which consists of lines selected from a wide range of genetic backgrounds. The higher diversity of CIMMYT germplasm vis-à-vis other wheat germplasm sets has also been observed in previous studies (Warburton et al., 2006; Dreisigacker et al., 2008; Sehgal et al., 2015).

We compared the stable haplotypes identified in the our study with GWAS peaks for GY and yield-related traits identified in various other panels using the GrainGenes genome browser^1^. Additionally, we investigated overlaps of the stable haplotypes against the meta-QTL (MQTL) reported by Acuña-Galindo et al. (2015) associated with adaptation to drought and heat stress (Supplementary Table 3). Furthermore, we compared our results with those reported by Li et al. (2019), who located 12 stable QTL for GY on the wheat reference genome using haplotype-based GWAS (Supplementary Table 4). Of the 7, 4, 10, and 15 environment-specific associations identified in the I, MD, SD, and HS environments, respectively, the four associations identified in the MD environment corresponded to MQTL 2, 6, 13, and 27. One (HB5.6) and three (HB5B.16, HB7A.20, HB7A.32) haplotype blocks identified in the SD and HS environments, respectively, corresponded to MQTL 44, 58, and 59 of Acuña-Galindo et al. (2015). Further, two (HB3A.1 and HB6B.7), three (HB4A.20, HB5B.6, and HB6A.6), and two (HB3B.25 and HB7A.3) haplotype blocks identified in the I, SD, and HS environments, respectively, overlapped with known GY QTL in GrainGenes (Supplementary Table 3). Juliana et al. (2019) used single marker–based GWAS on a smaller subset (3,485 lines) of the same EYT investigated here. The authors reported QTL within 0.2–2.2 Mb of the stable haplotype blocks reported on chromosome 3B (HB3B.25) in the HS environment and on chromosome 4A (HB4A.23 and HB4A.24) in the I, SD, and HS environments. Other QTL reported by Juliana et al. (2019) were on the same chromosomes as the present study; however, these were 58–510 Mb apart. For instance, haplotype blocks identified on chromosomes 5B (HB 5B.21) and 6B (HB 6B.20) were 58 and 146 Mb apart, whereas the four haplotype blocks identified on chromosome 7B (HB7B.11, HB7B.18, HB7B.21, and HB7B.45) were 98, 353, 382, and 510 Mb apart, respectively. Most significantly in our study, a haplotype block hotspot region was identified on chromosome 7A for heat tolerance, which was not detected in previous studies.

Of the 30 stable haplotype blocks identified in multiple environments and across EYTs, six corresponded with GWAS peaks identified in GrainGenes, while eight blocks corresponded to five MQTL (MQTL2 covered by HB1A.12, HB1A.13, and HB1A.14; MQTL6 covered by HB1B.19 and 1B.20; MQTL27 covered by HB3B.2; MQTL44 covered by HB5B.21; and MQTL51 covered by HB6B.6) of Acuña-Galindo et al. (2015). When comparisons were made with the 12 stable QTL reported by Li et al. (2019) for GY and yield components, only two were found in close vicinity from 5 to 20 Mb (Supplementary Table 4).

The frequencies of the favorable haplotypes of the 30 stable multi-environmental haplotypes blocks revealed that eight favorable haplotypes in blocks HB1A.12, HB1B.3, HB1B.19, HB2B.42, HB4B.8, HB5B.3, HB6B.20, and HB7B.18 decreased slightly by 10–18% over the 7 years, and only one favorable haplotype in the block HB7B.21 showed a sharp decrease of 36% in the seventh year (EYT2017-18). Eleven favorable haplotypes were maintained in moderate (30–50%) frequencies. Intriguingly, favorable haplotype in the block HB5D.5, with an allelic effect of +116–496 kg/ha across environments, was maintained at the highest frequency (up to 77%) in all seven EYTs, whereas the frequencies of the seven favorable haplotypes in blocks HB1B.20, HB2B.10, HB3B.2, HB4A.20, HB4A.27, HB5A.15, and HB6B.38 remained consistently low (2–15%) across EYTs. These low-frequency haplotypes were significantly associated with GY in three or all four environments and showed moderate to high allelic effects varying from +85–233 kg/ha to +148–449 kg/ha across EYTs and hence are important targets for future validation.

Despite the awareness that epistasis contributes significantly to the genetic architecture of most quantitative traits, epistatic interactions are usually not explored in GWAS studies (Sehgal et al., 2017, 2020; Assefa et al., 2019). The most important reason is that it is time consuming and computationally exhaustive to estimate genome-wide interactions in large datasets. Further, unlike in bi-parental populations, ready-to-use models are not available to estimate marker interaction effects along with main additive effects in GWAS panels (Rio et al., 2020). Additionally, the lack of sufficiently large experimental datasets has been a limiting factor to obtain reasonable statistical power when screening the genome for multi-locus epistasis. The size of our GWAS panel (6,333 lines) in the present study, along with the comprehensive phenotypic datasets generated in multiple environments (irrigated and stress environments), in combination with the fact that a large single SNP dataset was reduced to a set with fewer haplotype blocks, made the study of multi-locus epistatic interactions feasible with reasonable statistical power. We observed significant interactions among stable haplotypes. Most importantly, the haplotype block HB5B.29 linked to the vernalization locus *Vrn-B1* seemed to contribute significantly to interactions in both irrigated and drought-stressed environments, explaining up to 12.9% additional variation (Supplementary Figures 4, 5). This reinforces that major flowering genes can contribute to yield advantage in both irrigated and drought-stressed environments by both additive and epistatic effects (Cockram et al., 2007; Sehgal et al., 2017; He et al., 2019).

Likewise, significant epistatic interactions were obtained among genome-wide haplotypes for GY, explaining a higher percentage of variation in severely stressed environments (SD and HS) compared to the I environment in all EYTs (Supplementary Figures 9, 10). Our results are in contrast to Reif et al. (2011), who reported that main effects dominated the genetic architecture of GY and epistatic interactions contributed only little. We attribute these discrepancies to a narrower panel of elite breeding lines (455 lines, derivatives from a few parents) used in Reif et al. (2011) that probably did not retain enough power to reveal epistasis among loci. Further, Reif et al. (2011) studied GY only in irrigated environments whereas in the current study multiple environments were analyzed.

To be able to utilize stable QTL in a breeding program, we constructed heat maps for all environments in all EYTs. This approach led us to recognize different sets of lines with contrasting haplotype composition. CIMMYT and other breeding programs start to routinely genotype all lines that enter yield trials. Therefore, lines with higher numbers of favorable haplotypes and complementary haplotypes can be identified and re-incorporated as parents in breeding programs to maintain and further accumulate the favorable haplotypes in subsequent breeding cycles. The results can also be exploited in multiple-trait integration or line-conversion pipelines by using the lines carrying high numbers of favorable haplotypes as elite parents in crosses with donor parents selected for additional target traits (e.g., disease resistance) to be able to reveal a comprehensive performance package. The latest studies have shown that integrating haplotypes and epistatic interactions as fixed effects in genome-wide prediction models can improve prediction abilities for GY by about 10% (Spindel et al., 2016; Sehgal et al., 2020). This approach that attempts to boost prediction abilities with the contribution of GWAS peaks has yet to be further tested. Further, Mérida-García et al. (2020) reported candidate genes underpinning metaQTL reported by Acuña-Galindo et al. (2015) on chromosomes 3B and 4A. The candidate genes reported here (Supplementary Table 6) with proven roles in abiotic stress tolerance in model crops or having expression evidences in wheat under various stress conditions expand opportunities for future validation studies (Mérida-García et al., 2020).

## Data Availability Statement

The data related to manuscript has been provided in Supplementary Material. The genotypic and phenotypic data are available at link: https://data.cimmyt.org/dataset.xhtml?persistentId=hdl:11529/10548504.

## Author Contributions

DS and SD conceptualized the manuscript. SD designed the research. DS analyzed the data and wrote the manuscript. SM, LC-H, GV, JH-E, and RS generated phenotypic data. PJ, SS, and JP provided allele called GBS data. All authors reviewed the manuscript.

## Conflict of Interest

The authors declare that the research was conducted in the absence of any commercial or financial relationships that could be construed as a potential conflict of interest.

## Footnotes

1 https://wheat.pw.usda.gov/GG3/genome_browser
