# Supplementary material for: Haplotype-Based, Genome-Wide Association Study Reveals Stable Genomic Regions for Grain Yield in CIMMYT Spring Bread Wheat
Source: Front Genet. 2020 Dec 3;11:589490. doi: 10.3389/fgene.2020.589490 (PMC7737720; doi:10.3389/fgene.2020.589490)
Supplement: Supplementary file 1 [file Data_Sheet_1.PDF]

Code for ANOVA

```
#work directory

setwd("Folder path")

#read file

set=read.csv("File name.csv")

set$Gen=as.factor(set$Gen)

set$Rep=as.factor(set$Rep)

str(set)

library(lme4)

install.packages("lme4")

m1=lm(GY~1+Env+Env:Rep+Gen+Env*Gen,data=set)

anova(m1)
```

Code for interactions

#The code is designed in such a way that it discriminates genome wide and associated markers in the genotypic file. For example, genome wide haplotypes labelled as M1, M2, …………..Mn and associated haplotypes are labelled as GeneM3, GeneM5…………………….GeneMn etc.

```
library(lattice)

library(coxme)

library(MASS)

#set work directory

setwd("Folder path")

#read phenotypic data

P<-read.table("Phenotypic data.txt",header=T, dec=",", sep="\t",stringsAsFactors=FALSE,na.string="NA")

P

#read.kinship

K<-read.table("kinship.txt",row.names=1, dec=".", sep="\t",stringsAsFactors=FALSE)

colnames(K)<-row.names(K)

K<-as.matrix(K)
```

```
K

#read structure
Q<-read.table("PCs.txt",header=T, dec=".", sep="\t",stringsAsFactors=FALSE)
Q
#read genotyping data and contain only the useful markers
G<-read.table("genotypic.txt",header=T, dec=".", sep="\t",stringsAsFactors=FALSE)
G

#on how many trait we will work?, change if necessary
nb_trait<-1
trait<-"Trait5"
nb_trait

#How many associated markers (Genes)?
#If only the one which starts by "gene"
gene_list<-colnames(G)[grep("Gene",colnames(G)[-1])]
gene_list

#Manual way
#gene_list or associated markers<-c("M12","M4","M3")
nb_gene<-nlevels(as.factor(gene_list))
nb_gene

#How many PCA axes are there in the Q file?
nb_PCA<-dim(Q)[2]-1
nb_PCA

#How many axes you will use?
```

```
PCA_use<-3 #be sure to put as much in the asreml model
RESULTAT_F<-0
for (i in 1:nb_trait)
{

 #nb_marker_ok<-nlevels(as.factor(Marker_OK[which(Marker_OK$Trait==trait[i]),2]))
 #marker_ok<-levels(as.factor(Marker_OK[which(Marker_OK$Trait==trait[i]),2]))
 nb_marker_ok<-dim(G)[2]-1
 marker_ok<-colnames(G)[-1]

 a<-0

 for (j in 1:nb_marker_ok)
 {

  for (k in 1:nb_gene)
  {
   print(c("trait",i,"on",nb_trait))
   a<-a+1
   print(c(a,"on",nb_marker_ok*nb_gene))

   #Prepare a file with all information for a combination marker_gene
   GWAS<-merge(Q,P[,c(1,which(colnames(P)==trait[i]))],by="LINE")
   GWAS<-merge(GWAS,G[,c(1,which(colnames(G)==marker_ok[j])
                 ,which(colnames(G)==gene_list[k]))],by="LINE")

   GWAS<-GWAS[which(is.na(GWAS[,nb_PCA+3])==F),]
   GWAS<-GWAS[which(is.na(GWAS[,nb_PCA+4])==F),]
```

```
GWAS[,nb_PCA+5]<-paste(GWAS[,nb_PCA+3],GWAS[,nb_PCA+4])
GWAS[,nb_PCA+5]<-as.factor(GWAS[,nb_PCA+5])
GWAS[,nb_PCA+4]<-as.factor(GWAS[,nb_PCA+4])
GWAS[,nb_PCA+3]<-as.factor(GWAS[,nb_PCA+3])
GWAS[,1]<-as.factor(GWAS[,1])
colnames(GWAS)[nb_PCA+5]<-"MARKER_GENE"
colnames(GWAS)[nb_PCA+4]<-"GENE"
colnames(GWAS)[nb_PCA+3]<-"MARKER"
colnames(GWAS)[nb_PCA+2]<-"TRAIT"
resum<-as.data.frame(table(GWAS[,nb_PCA+5]))

if(dim(resum)[1]!=4) next

#Set up kinship matrix
K1<-K[which(row.names(K) %in% levels(GWAS[,"LINE"]))
     ,which(colnames(K) %in% levels(GWAS[,"LINE"]))]

############
#only I change this because appears like character and should be numeric
GWAS$TRAIT=as.numeric(GWAS$TRAIT)
###############

###Association
#Make sure how have the same number of pca axes that in PCA_use
mm <-lmekin(TRAIT ~ PC1+PC2+PC3+MARKER+GENE+MARKER:GENE+(1|LINE),
          varlist=list(K1),data=GWAS)

z <- fixef(mm) / sqrt(diag(vcov(mm, useScale = FALSE)))
Pvalue <- 2 * (1 - pnorm(abs(z)))
```

```
      #Extract coefficients
      COEFF<-fixef(mm)[(PCA_use+2):(PCA_use+4)]

      #Number of lines per allele combinations
      EFF<-cbind(t(data.frame(resum[,1])),t(data.frame(resum[,2])))[1,]

      SNP1<-marker_ok[j]
      SNP2<-gene_list[k]

      print(c(SNP1,SNP2))

      #P-value
      PVAL<-Pvalue[(PCA_use+2):(PCA_use+4)]

      #set results table
      RESULTAT<-c(trait[i],SNP1,SNP2,EFF,PVAL,COEFF)
      RESULTAT_F<-rbind(RESULTAT_F,RESULTAT)

    }
  }
}

colnames(RESULTAT_F)<-c("TRAIT","Marker","GENE","M_ALL1/G_ALL1","M_ALL1/G_ALL2",
                    "M_ALL2/G_ALL1","M_ALL2/G_ALL2","NB1","NB2","NB3","NB4","PVAL_M",
                    "PVAL_G","PVAL_M:G","coeff(M_ALL2)","coeff(G_ALL2)","coeff(M_ALL2/G_ALL2)")

write.table(RESULTAT_F,file="MARKER_GENE_INTERACTION.txt",sep="\t",dec=".",row.names=F)
```
