# Supplementary figures and images for: Haplotype-Based, Genome-Wide Association Study Reveals Stable Genomic Regions for Grain Yield in CIMMYT Spring Bread Wheat
Source: Front Genet. 2020 Dec 3;11:589490. doi: 10.3389/fgene.2020.589490 (PMC7737720; doi:10.3389/fgene.2020.589490)

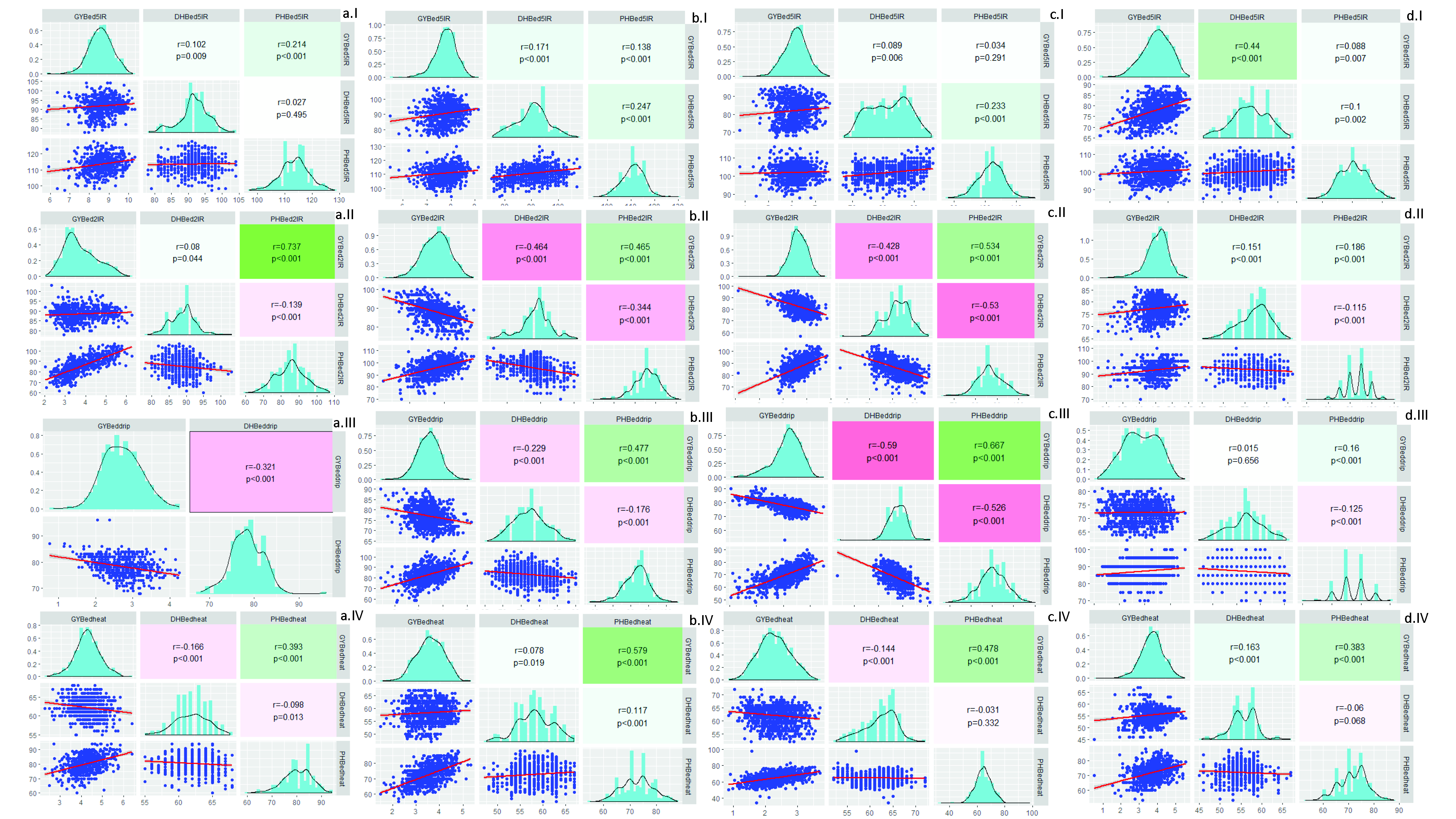

Supplement: Supplementary file 2 [file Image_1.TIFF]

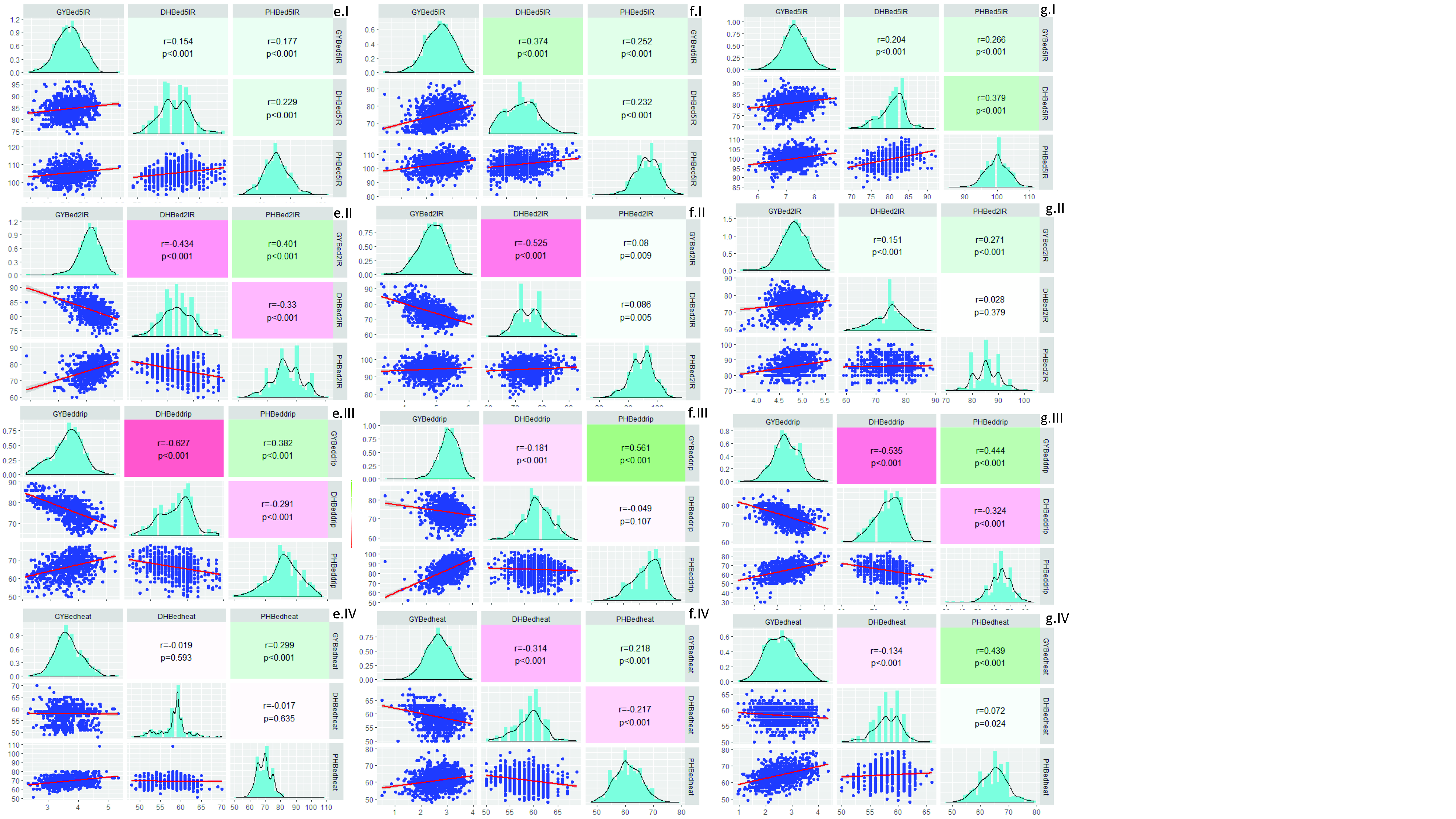

Supplement: Supplementary file 3 [file Image_2.TIF]

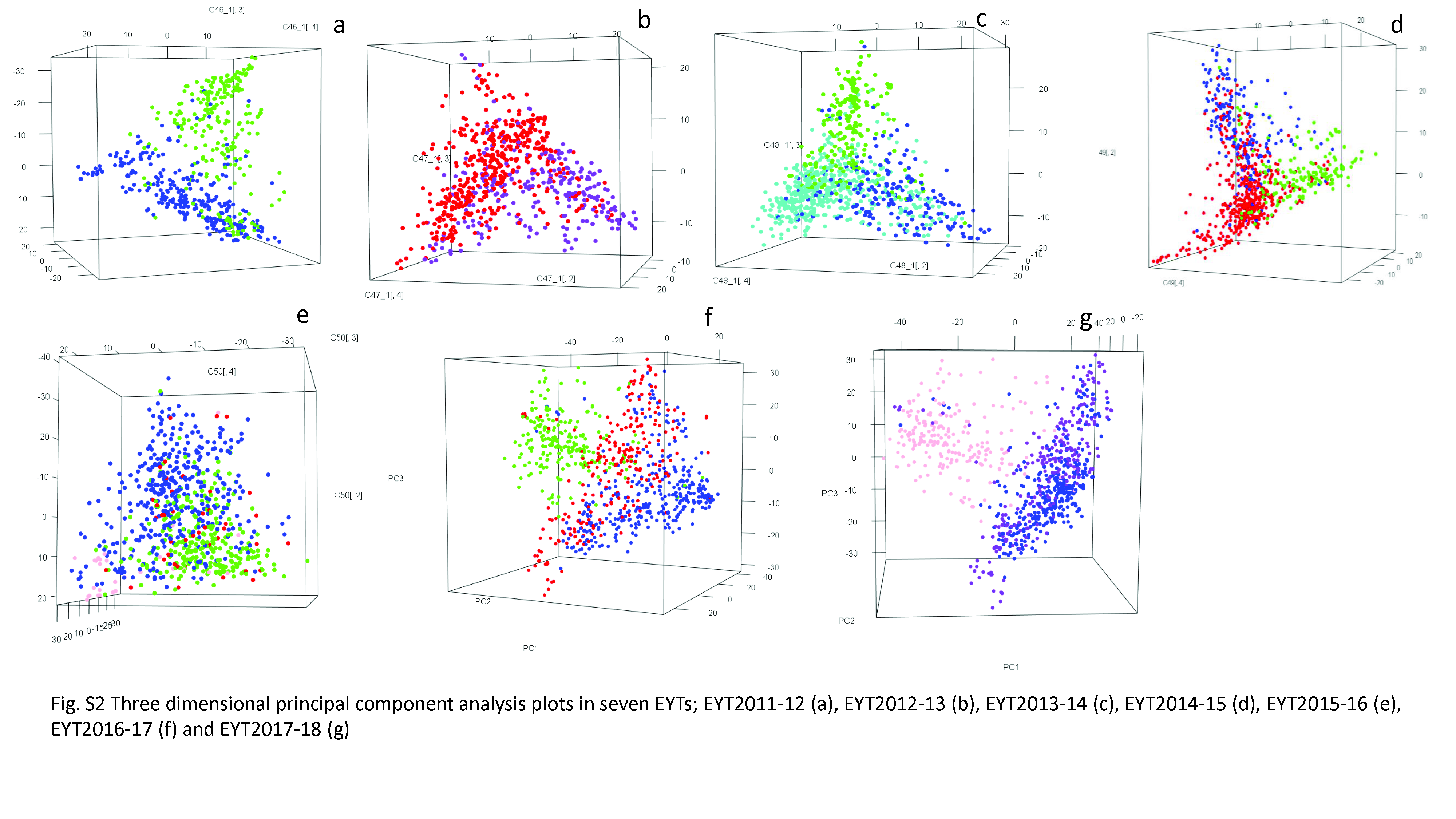

Supplement: Supplementary file 4 [file Image_3.TIF]

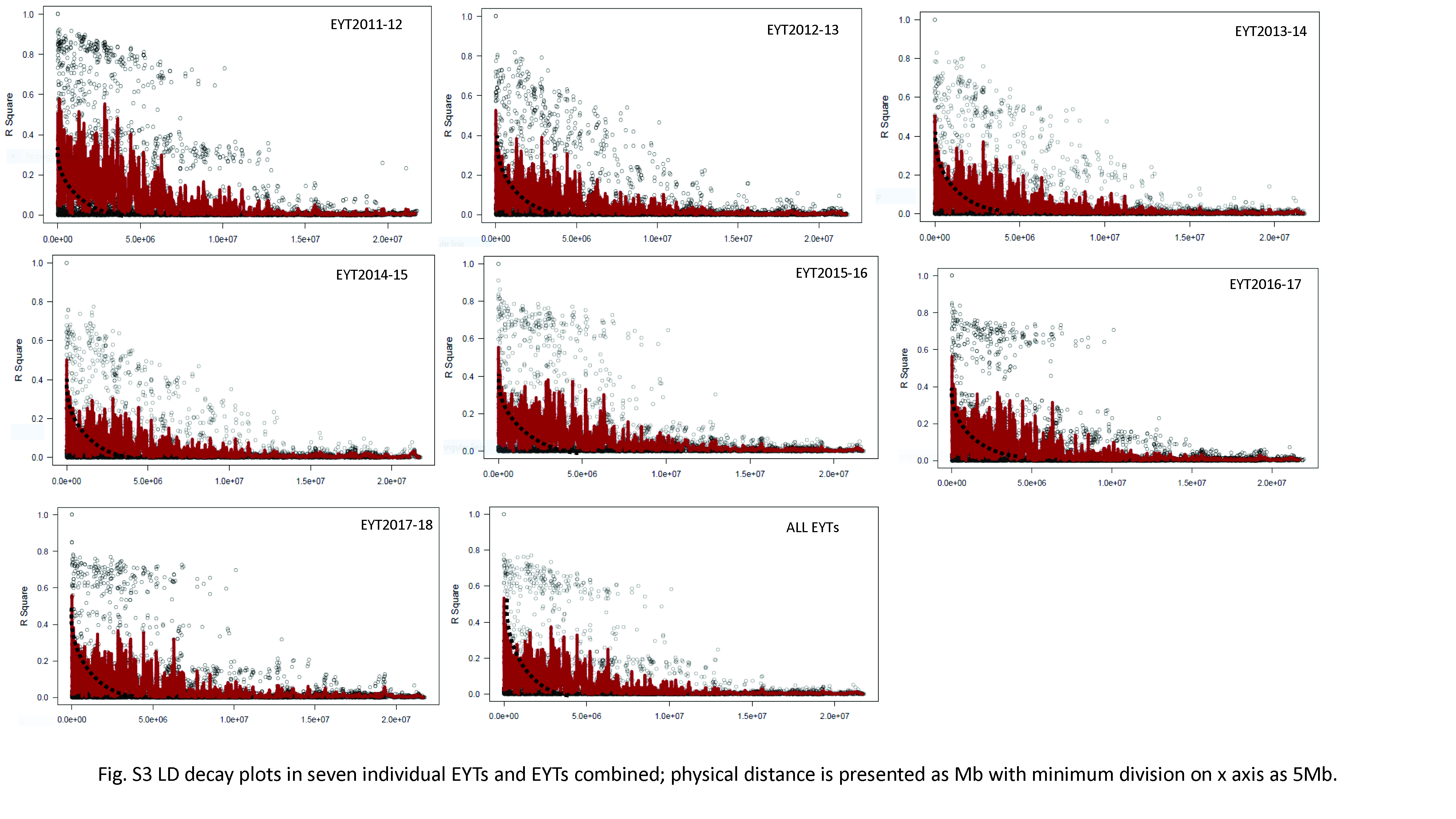

Supplement: Supplementary file 5 [file Image_4.TIF]

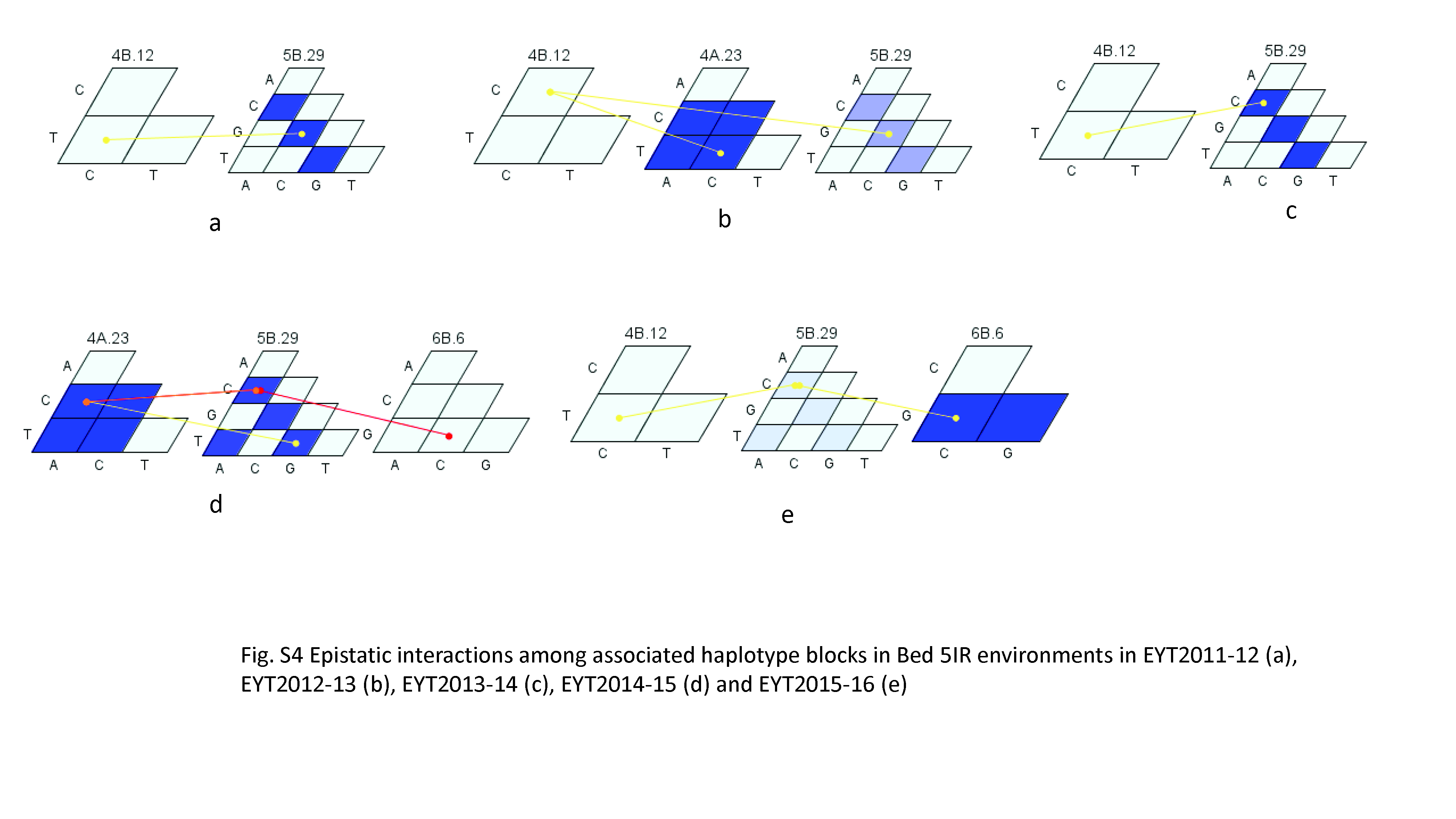

Supplement: Supplementary file 6 [file Image_5.TIF]

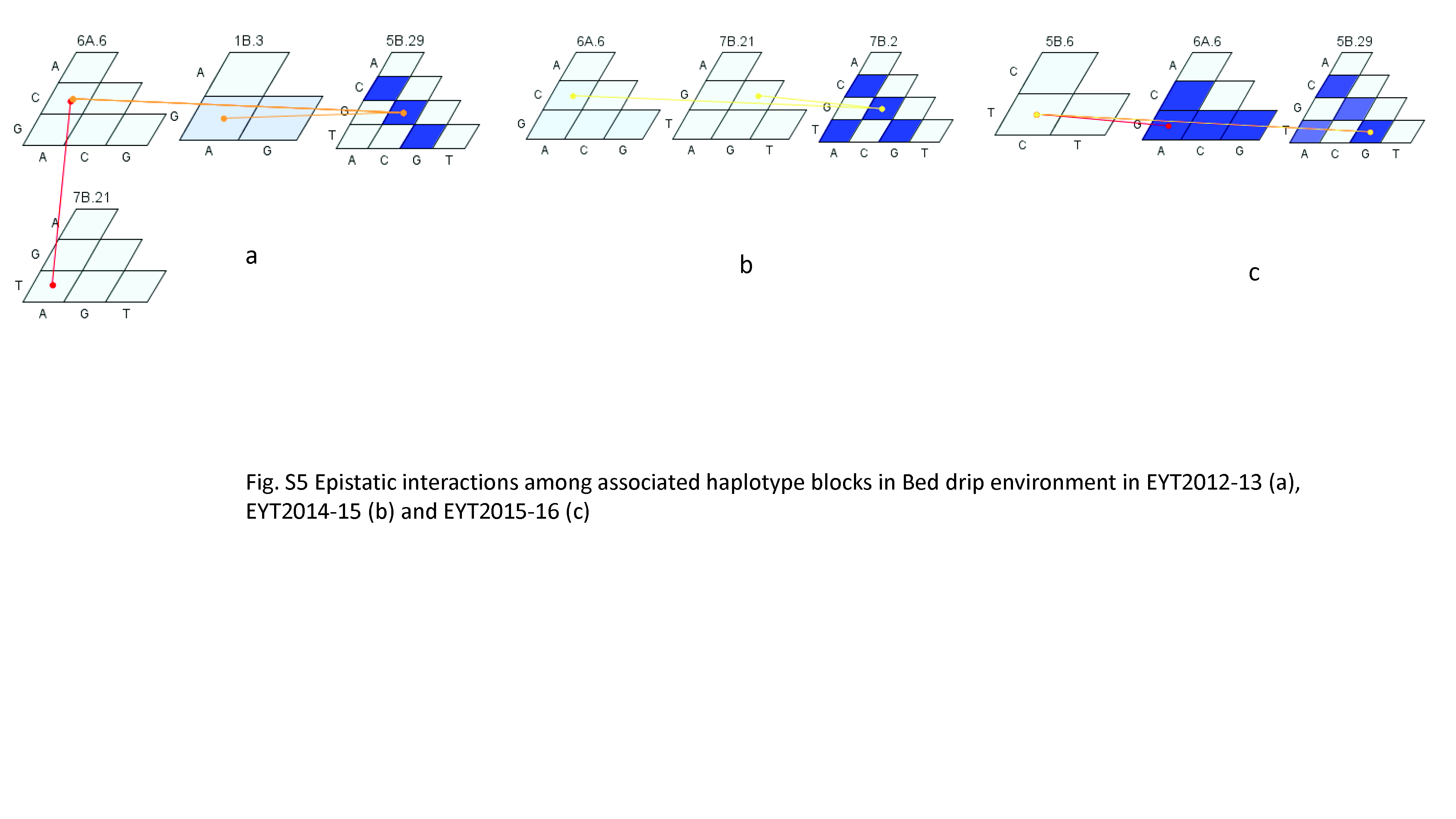

Supplement: Supplementary file 7 [file Image_6.TIF]

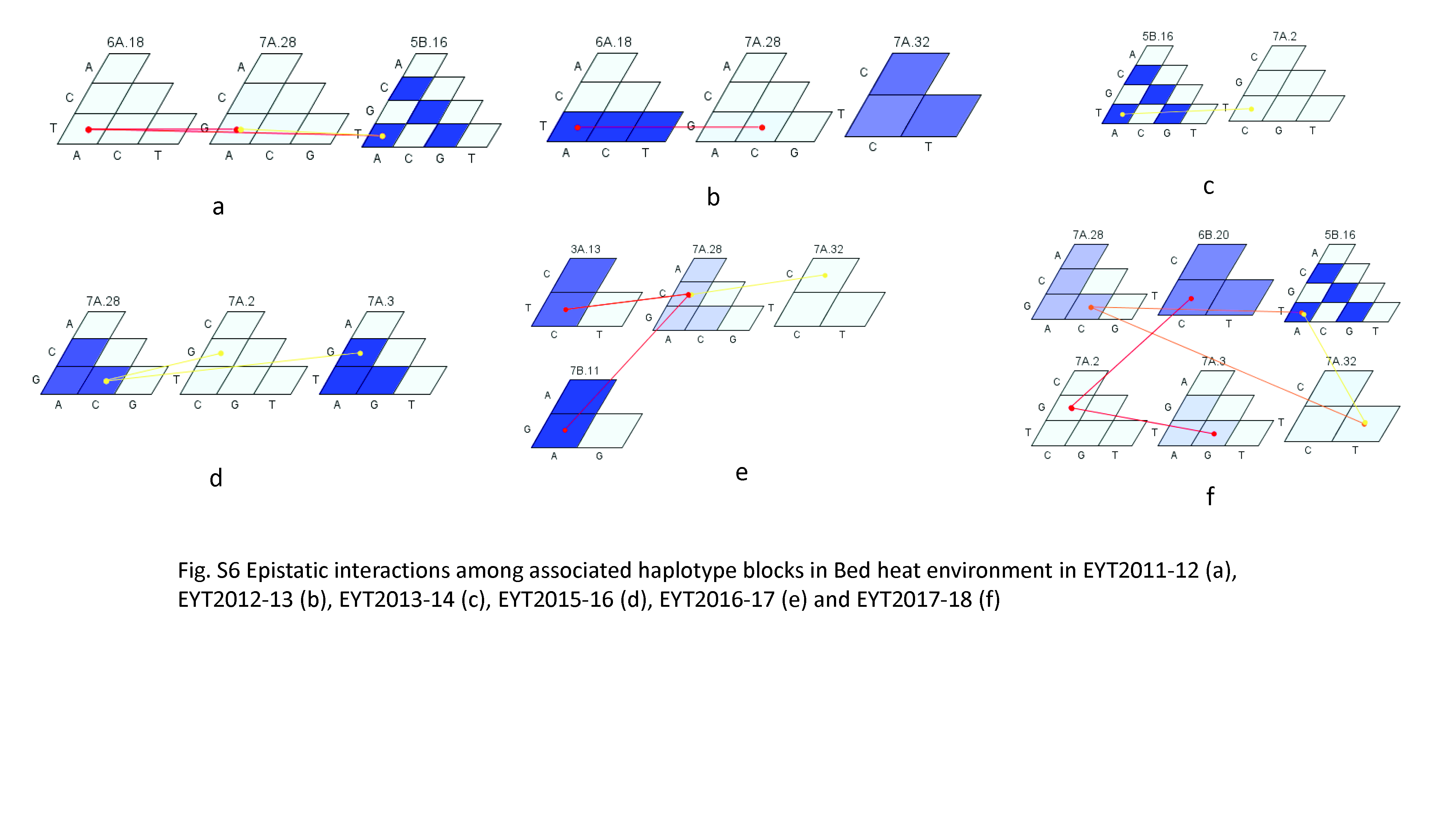

Supplement: Supplementary file 8 [file Image_7.TIF]

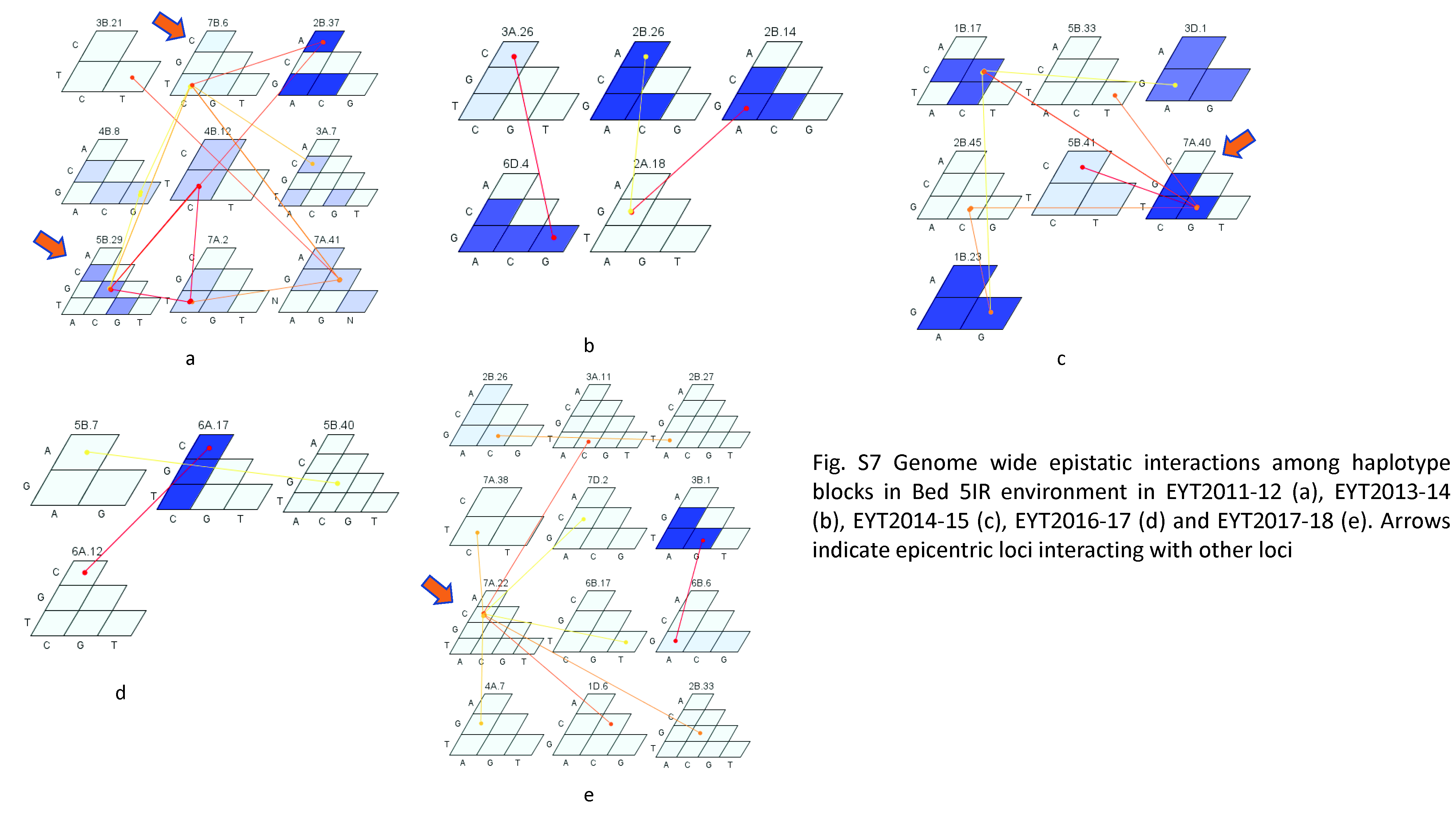

Supplement: Supplementary file 9 [file Image_8.TIF]

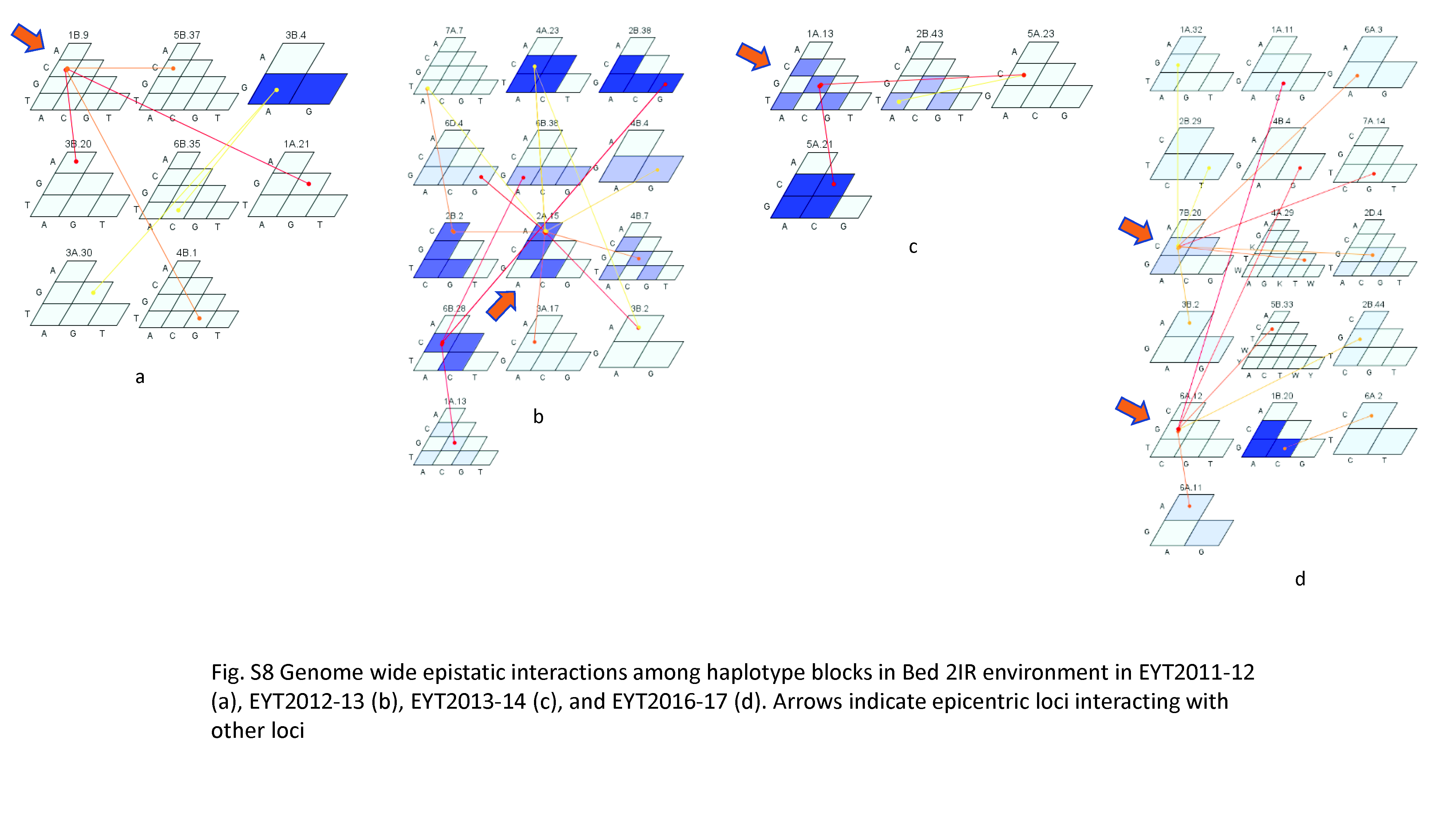

Supplement: Supplementary file 10 [file Image_9.TIF]

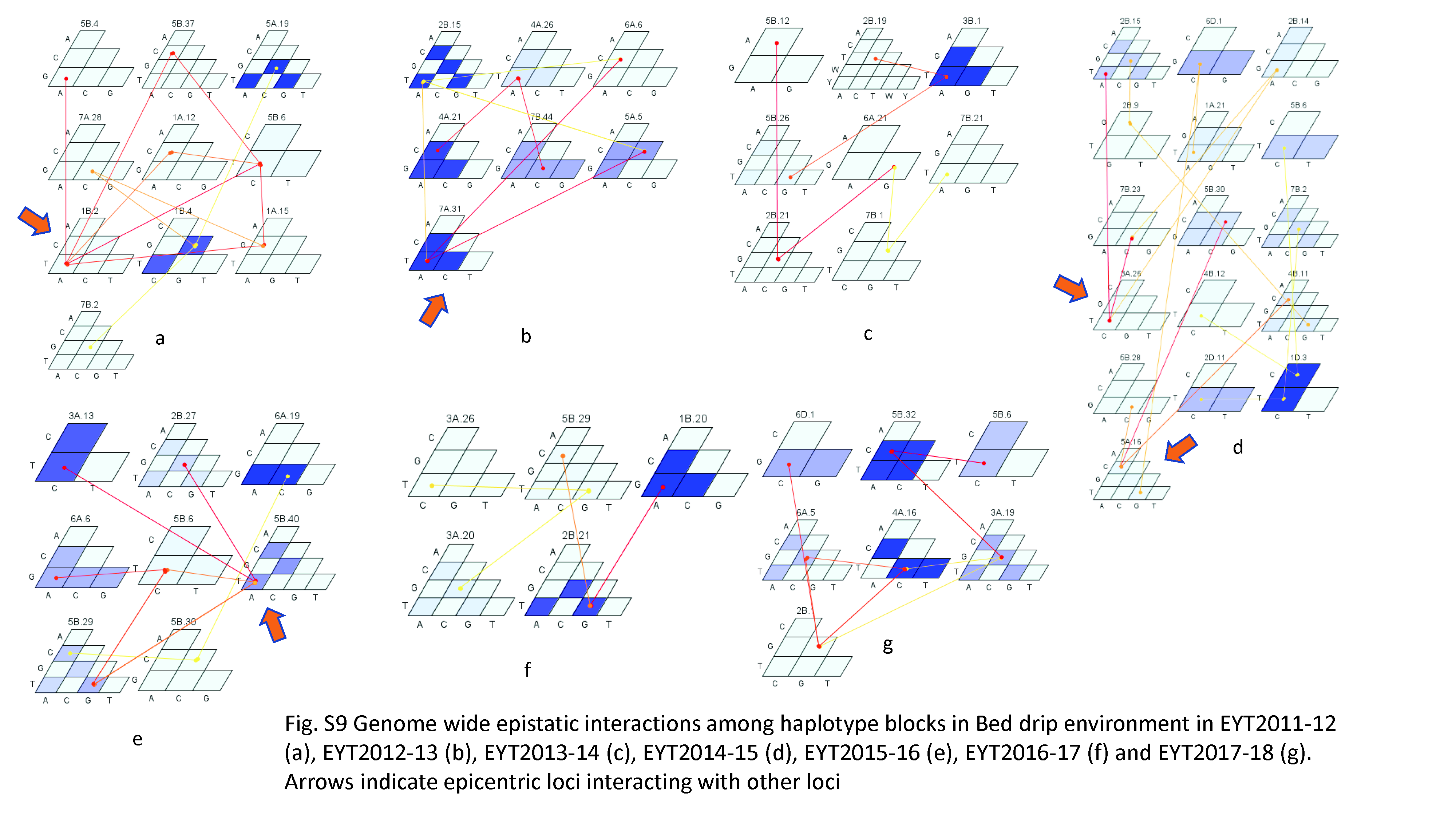

Supplement: Supplementary file 11 [file Image_10.TIF]

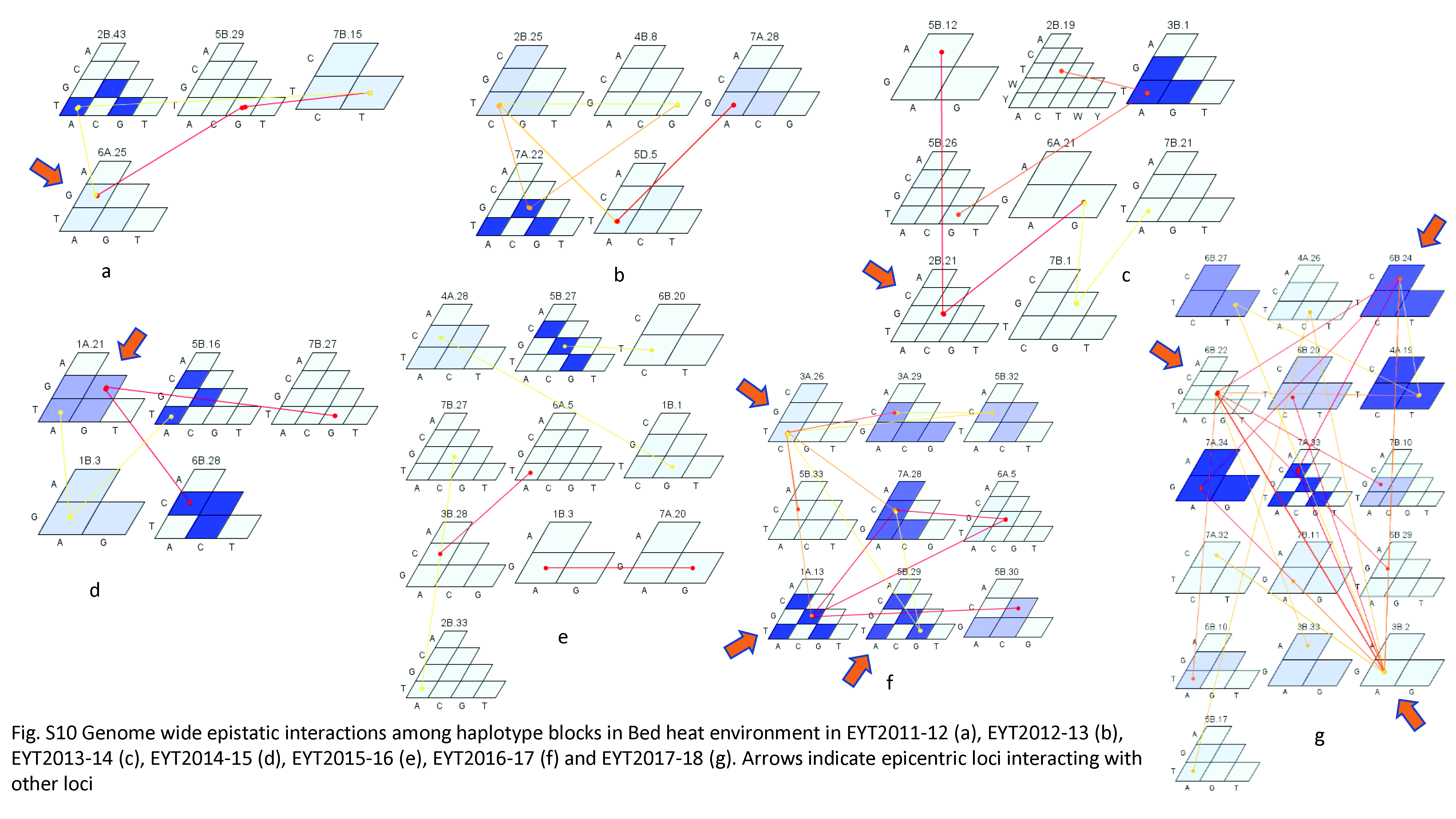

Supplement: Supplementary file 12 [file Image_11.TIFF]
