# Supplementary material for: Haplotype-Based, Genome-Wide Association Study Reveals Stable Genomic Regions for Grain Yield in CIMMYT Spring Bread Wheat
Source: Front Genet. 2020 Dec 3;11:589490. doi: 10.3389/fgene.2020.589490 (PMC7737720; doi:10.3389/fgene.2020.589490)
Supplement: Supplementary file 14 [file Table_2.DOCX]

Table S2. Adjusted mean of GY, ANOVA and across environment heritability (*h^2^*) of GY in the seven elite yield trails (EYT)

|  |  | Mean GY (kg/ha) | | |  |  |  |  |  |
| --- | --- | --- | --- | --- | --- | --- | --- | --- | --- |
|  | I | MD | SD | HS | ANOVA | | | | *h^2^* (across envs.) |
|  |  |  |  |  |  | Df | F value | Pr (>F) |  |
| EYT2011-12 | 8622±631 | 3763±834 | 2662±532 | 4275±605 | Rep | 2 | 2.3662 | 0.09394 | 0.63 |
|  |  |  |  |  | Geno | 642 | 10.6791 | p<0.0001 |  |
|  |  |  |  |  | Env | 3 | 61889.3558 | p<0.0001 |  |
|  |  |  |  |  | Env:Rep | 8 | 3.5718 | p<0.0001 |  |
|  |  |  |  |  | Geno:Env | 1926 | 4.9745 | p<0.0001 |  |
|  |  |  |  |  | Residuals | 5141 |  |  |  |
| EYT2012-13 | 7737±450 | 4741±403 | 3438±507 | 3576±590 | Rep | 2 | 5.3832 | p<0.01 | 0.32 |
|  |  |  |  |  | Geno | 900 | 9.4432 | p<0.001 |  |
|  |  |  |  |  | Env | 3 | 115410 | p<0.001 |  |
|  |  |  |  |  | Env:Rep | 8 | 5.441 | p<0.0001 |  |
|  |  |  |  |  | Geno:Env | 2712 | 7.094 | p<0.001 |  |
|  |  |  |  |  | Residuals | 7235 |  |  |  |
| EYT2013-14 | 6105±512 | 3698±388 | 2107±529 | 2305±504 | Rep | 2 | 19.098 | p<0.001 | 0.51 |
|  |  |  |  |  | Geno | 973 | 10.527 | p<0.001 |  |
|  |  |  |  |  | Env | 3 | 105510 | p<0.001 |  |
|  |  |  |  |  | Env:Rep | 8 | 17.322 | p<0.001 |  |
|  |  |  |  |  | Geno:Env | 2945 | 5.9152 | p<0.001 |  |
|  |  |  |  |  | Residuals | 7856 |  |  |  |
| EYT2014-15 | 5581±493 | 4541±369 | 2797±704 | 3735±641 | Rep | 2 | 9.2848 | p<0.001 | 0.57 |
|  |  |  |  |  | Geno | 928 | 14.1487 | p<0.001 |  |
|  |  |  |  |  | Env | 3 | 38863.7795 | p<0.001 |  |
|  |  |  |  |  | Env:Rep | 8 | 10.3116 | p<0.001 |  |
|  |  |  |  |  | Geno:Env | 2823 | 8.0261 | p<0.001 |  |
|  |  |  |  |  | Residuals | 7533 |  |  |  |
| EYT2015-16 | 7096±365 | 3201±392 | 1622±556 | 3628±443 | Rep | 2 | 11.457 | p<0.001 | 0.31 |
|  |  |  |  |  | Geno | 819 | 7.8758 | p<0.001 |  |
|  |  |  |  |  | Env | 3 | 127180 | p<0.001 |  |
|  |  |  |  |  | Env:Rep | 8 | 5.167 | p<0.001 |  |
|  |  |  |  |  | Geno:Env | 2483 | 4.9498 | p<0.001 |  |
|  |  |  |  |  | Residuals | 6589 |  |  |  |
| EYT2016-17 | 6500±528 | 4727±409 | 4089±396 | 2696±497 | Rep | 2 | 2.6029 | 0.07412 | 0.52 |
|  |  |  |  |  | Geno | 1085 | 10.692 | p<0.001 |  |
|  |  |  |  |  | Env | 3 | 89955.014 | p<0.001 |  |
|  |  |  |  |  | Env:Rep | 8 | 3.0916 | p<0.001 |  |
|  |  |  |  |  | Geno:Env | 3255 | 6.0959 | p<0.001 |  |
|  |  |  |  |  | Residuals | 8627 |  |  |  |
|  |  |  |  |  |  |  |  |  |  |
|  | I | MD | SD | HS |  | ANOVA |  |  | *h^2^* (across envs.) |
|  |  |  |  |  |  |  |  |  |  |
| EYT2017-18 | 7169±419 | 4759±293 | 2367±611 | 2562±497 | Rep | 2 | 43.616 | p<0.001 | 0.49 |
|  |  |  |  |  | Geno | 979 | 10.103 | p<0.001 |  |
|  |  |  |  |  | Env | 3 | 150640 | p<0.001 |  |
|  |  |  |  |  | Env:Rep | 8 | 26.755 | p<0.001 |  |
|  |  |  |  |  | Geno:Env | 2937 | 6.0169 | p<0.001 |  |
|  |  |  |  |  | Residuals | 7691 |  |  |  |
