# Supplementary material for: Haplotype-Based, Genome-Wide Association Study Reveals Stable Genomic Regions for Grain Yield in CIMMYT Spring Bread Wheat
Source: Front Genet. 2020 Dec 3;11:589490. doi: 10.3389/fgene.2020.589490 (PMC7737720; doi:10.3389/fgene.2020.589490)
Supplement: Supplementary file 15 [file Table_3.DOCX]

Table S3 Stable genomic regions associated with grain yield in elite yield trials (EYT) specific to four testing environments

| Testing env. (ID) | Hap. block | Markers in Hap. block | Chr | Interval; First-Last SNP (bp) | Haplotype alleles | Fav. allele | EYT | Allelic effect  (kg/ha) | Overlapping GWAS peaks for yield related traits (panels in GrainGenes database) | Overlap with meta-QTL |
| --- | --- | --- | --- | --- | --- | --- | --- | --- | --- | --- |
| Irrigated (Bed 5IR) | HB2A.13 | S2A_691480248,  S2A_691480277, S2A_691529982, S2A_691531800, S2A_691845674 | 2A | 365426 | CCCTA, CTCTG | CCCTA | EYT2011-12, EYT2012-13, EYT2013-14, EYT2014-15 | +30-286 |  |  |
|  | HB3A.1 | S3A_13375958,  S3A_13376453,  S3A_13376456,  S3A_13496895 | 3A | 120937 | ACGA,  GCCG,  GTCG | ACGA | EYT2013-14, EYT2014-15, EYT2016-17 | +215-525 | TCAP spring wheat AM panel |  |
|  | HB4A.23 | S4A_719934007,  S4A_719951086 | 4A | 17079 | AC, CT | CT | EYT2013-14, EYT2015-16, EYT2016-17 | +71-112 |  |  |
|  | HB4B.12 | S4B_663621978,  S4B_663622013 | 4B | 35 | CC, CT, TC | TC | EYT2013-14, EYT2014-15, EYT2016-17 | +168-429 |  |  |
|  | HB5B.29 | S5B_557138172,  S5B_557138254 | 5B | 82 | AC, GC, GT | AC, GT | EYT2011-12, EYT2012-13, EYT2013-14 | +47-568 |  |  |
|  | HB6B.6 | S6B_17686703,  S6B_17701765 | 6B | 15062 | AG, GC | GC | EYT2011-12, EYT2014-15, EYT2016-17, EYT2017-18 | +62-299 |  |  |
|  | HB6B.7 | S6B_18241052,  S6B_18432828,  S6B_18592415,  S6B_18595727,  S6B_18730568 | 6B | 489516 | GTCTG,  TCAGA,  TCAGG | GTCTG | EYT2011-12, EYT2013-14, EYT2016-17 | +76-492 | Elite HWWAM Panel NUE, TCAP NUE Elite Hard Winter Wheat AM Panel |  |
| Moderate drought (Bed 2IR) | HB1A.13 | S1A_497201550,  S1A_497201682 | 1A | 132 | CG, TA | CG | EYT2012-13, EYT2013-14, EYT2016-17 | +100-174 |  | MetaQTL-2 |
|  | HB1B.19 | S1B_639415604,  S1B_639415692,  S1B_639426265 | 1B | 10661 | CCG, GTA,  GTG | CCG | EYT2011-12, EYT2013-14, EYT2014-15  EYT2015-16 | +92-553 |  | MetaQTL-6 |
|  | HB2A.29 | S2A_768927927,  S2A_768927939,  S2A_768930701 | 2A | 2774 | CAA,  GAC,  GTC | CAA | EYT2011-12, EYT2012-13, EYT2014-15  EYT2015-16 | +86-206 |  | MetaQTL-13 |
| Testing env. (ID) | Hap. block | Markers in Hap. block | Chr | Interval; First-Last SNP (bp) | Haplotype alleles | Fav. allele | EYT | Allelic effect | Overlapping GWAS peaks for yield related traits (panels in GrainGenes database) | Overlap with meta-QTL |
|  | HB3B.2 | S3B_7240747,  S3B_7240753 | 3B | 6 | AA, AG, GG | GG | EYT2014-15, EYT2016-17, EYT2017-18 | +53-248 |  | MetaQTL-27 |
| Severe drought (Bed drip) | HB1A.29 | S1A_585645864,  S1A_585645875,  S1A_585656529,  S1A_585656558 | 1A | 10694 | AAAC, GAGC | AAAC | EYT2011-12, EYT2012-13, EYT2013-14 | +61-161 |  |  |
|  | HB1B.3 | S1B_18569448,  S1B_18570787 | 1B | 1339 | AG, GA, GG | GG, AG | EYT2013-14, EYT2015-16, EYT2017-18 | +126-359 |  |  |
|  | HB4A.20 | S4A_713064971,  S4A_713506269,  S4A_713517340,  S4A_713522176 | 4A | 457205 | CGTC, TACT,  TGTC | CGTC | EYT2011-12, EYT2013-14, EYT2017-18 | +133-233 | TCAP WUE Hard Winter Wheat AM Panel, TCAP NUE WUE Soft Winter Wheat Panel |  |
|  | HB4A.24 | S4A_720045719,  S4A_720047771,  S4A_720048088 | 4A | 2369 | ACT,  GTC | ACT | EYT2011-12, EYT2012-13, EYT2016-17 | +73-129 |  |  |
|  | HB4D.1 | S4D_455090934,  S4D_455321401,  S4D_455349432,  S4D_455472313 | 4D | 381379 | CTTG,  TCCA,  TCTG | TCTG | EYT2013-14, EYT2015-16, EYT2016-17 | +151-362 |  |  |
|  | HB5B.29 | S5B_557138172,  S5B_557138254 | 5B | 82 | AC, GC,  GT | GT | EYT2012-13, EYT2013-14, EYT2015-16, EYT2016-17, EYT2017-18 | +263-430 |  |  |
|  | HB5B.6 | S5B_47584429,  S5B_47592949 | 5B | 8520 | CT, TC | CT | EYT2012-13, EYT2013-14, EYT2014-15 | +127-250 | Spring Wheat AM Panel, TCAP WUE Hard Winter Wheat AM Panel | MetaQTL-44 |
|  | HB6A.6 | S6A_9321817,  S6A_9339844 | 6A | 18027 | CA, CG, GA | GA | EYT2013-14, EYT2015-16, EYT2016-17 | +203-248 | TCAP NUE WUE Soft Winter Wheat Panel, TCAP spring wheat AM panel |  |
|  | HB7B.2 | S7B_18097645,  S7B_18097824 | 7B | 179 | AT, GC, GT | AT | EYT2012-13, EYT2013-14, EYT2017-18 | +85-237 |  |  |
|  | HB7B.21 | S7B_605313385,  S7B_605313397 | 7B | 12 | AT, GG,  GT | AT | EYT2011-12, EYT2012-13, EYT2017-18 | +105-296 |  |  |
| Heat stress (Bed heat) | HB2B.12 | S2B_50741298,  S2B_50741368,  S2B_51077637,  S2B_51227491 | 2B | 486193 | CCCG,  GGTA,  GGTG | GGTA | EYT2012-13, EYT2013-14, EYT2017-18 | +116-373 |  |  |
| Testing env. (ID) | Hap. block | Markers in Hap. block | Chr | Interval; First-Last SNP (bp) | Haplotype alleles | Fav. allele | EYT | Allelic effect | Overlapping GWAS peaks for yield related traits (panels in GrainGenes database) | Overlap with meta-QTL |
|  | HB3A.13 | S3A_143491853,  S3A_143493411 | 3A | 1558 | CT, TC | TC | EYT2012-13, EYT2013-14, EYT2015-16 | +30-202 |  |  |
|  | HB3B.1 | S3B_6085798,  S3B_6085816 | 3B | 18 | AT, GA, GT | GT | EYT2013-14, EYT2015-16, EYT2017-18 | +96-231 |  |  |
|  | HB3B.25 | S3B_762639074,  S3B_762743699,  S3B_763055468,  S3B_763055485 | 3B | 416411 | AAAA, AAAG, GTGG | GTGG | EYT2011-12, EYT2012-13, EYT2016-17 | +95-280 | Elite Hard Winter Wheat AM Panel |  |
|  | HB4A.24 | S4A_720045719,  S4A_720047771,  S4A_720048088 | 4A | 2369 | ACT, GTC | GTC | EYT2012-13, EYT2014-15, EYT2016-17 | +192-210 |  |  |
|  | HB5B.16 | S5B_455978226,  S5B_455980623 | 5B | 2397 | CG, TA | CG | EYT2012-13, EYT2013-14, EYT2017-18 | +81-164 |  | MetaQTL-44 |
|  | HB6A.18 | S6A_416649831,  S6A_416657252 | 6A | 7421 | AT, TC | TC | EYT2011-12, EYT2012-13, EYT2015-16 | +68-227 |  |  |
|  | HB6B.20 | S6B_459374225,  S6B_459374299 | 6B | 74 | CT, TC | CT | EYT2011-12, EYT2012-13, EYT2017-18 | +53-183 |  |  |
|  | HB6B.38 | S6B_708712113,  S6B_708712131 | 6B | 18 | GG, CG, GA | CG | EYT2013-14, EYT2014-15, EYT2017-18 | +113-265 |  |  |
|  | HB7A.2 | S7A_7938818,  S7A_7938819 | 7A | 1 | CT, GC, GT | GC | EYT2012-13, EYT2013-14, EYT2014-15, EYT2017-18 | +106-236 |  |  |
|  | HB7A.20 | S7A_116622729,  S7A_116626028 | 7A | 3299 | AA, GG | GG | EYT2012-13, EYT2013-14, EYT2015-16 | +179-236 |  | MetaQTL-58 |
|  | HB7A.28 | S7A_567030080,  S7A_567030245 | 7A | 165 | AG, CA,  CG | CA | EYT2013-14, EYT2015-16, EYT2016-17 | +221-255 |  |  |
|  | HB7A.3 | S7A_12011058,  S7A_12011069 | 7A | 11 | AT, GA, GT | AT | EYT2011-12, EYT2013-14, EYT2015-16, EYT2017-18 | +104-199 | TCAP WUE Hard Winter Wheat AM Panel |  |
|  | HB7A.32 | S7A_615374725,  S7A_615374790 | 7A | 65 | CC, TT | CC | EYT2012-13, EYT2015-16, EYT2017-18 | +230-416 |  | MetaQTL-59 |
|  | HB7B.11 | S7B_124548883,  S7B_124549059 | 7B | 176 | AG, GA | AG | EYT2011-12, EYT2012-13, EYT2014-15, EYT2017-18 | +69-267 |  |  |
