# Supplementary material for: Haplotype-Based, Genome-Wide Association Study Reveals Stable Genomic Regions for Grain Yield in CIMMYT Spring Bread Wheat
Source: Front Genet. 2020 Dec 3;11:589490. doi: 10.3389/fgene.2020.589490 (PMC7737720; doi:10.3389/fgene.2020.589490)
Supplement: Supplementary file 16 [file Table_4.DOCX]

Table S4 Thirty stable associations identified for grain yield considering all EYT and four testing environments (Irrigated; I, Moderate drought; MD, Severe Drought; SD, Heat Stress; HS) together

| Hap.  block | Markers in Hap. block | Chr | Interval; First-Last SNP (bp) | Haplotype alleles | Fav. allele | EYT; Testing environment | Allelic effect (kg/ha) | Overlapping GWAS peaks of yield related traits (panels in GrainGenes database) | Overlap with meta-QTL | Overlap with /distance from haplotypes-based stable QTL (Li et al. 2019) |
| --- | --- | --- | --- | --- | --- | --- | --- | --- | --- | --- |
| HB1A.12 | S1A_494392059,  S1A_494393037 | 1A | 978 | CA, GG | CA | EYT2011-12; DS, EYT2012-13; HS, EYT2013-14; MD, EYT2013-14; SD, EYT2016-17; MD | +44-258 |  | MetaQTL-2 | 49Mb from stable QTL |
| HB1A.13 | S1A_497201550,  S1A_497201682 | 1A | 132 | CG, TA | CG | EYT2012-13; MD, EYT2013-14; MD, EYT2013-14; SD, EYT2016-17; MD, EYT2016-17; HS | +100-188 |  | MetaQTL-2 | 52Mb from stable QTL |
| HB1A.14 | S1A_499864157,  S1A_499864420,  S1A_499864432,  S1A_500074551 | 1A | 210394 | CCGC, TGCT | TGCT | EYT2011-12; MD, EYT2012-13; HS, EYT2015-16; MD, EYT2016-17; MD, EYT2016-17; HS | +35-235 | Spring Wheat AM Panel | MetaQTL-2 | 54Mb from stable QTL |
| HB1B.3 | S1B_18569448,  S1B_18570787 | 1B | 1339 | AG, GA | AG | EYT2011-12; I, EYT2013-14; SD, EYT2014-15; MD, EYT2014-15; HS, EYT2015-16; SD, EYT2017-18; SD | +88-351 |  |  | 354 – 488 Mlb from stable QTL |
| HB1B.19 | S1B_639415604,  S1B_639415692,  S1B_639426265 | 1B | 10661 | CCG, GTA,  GTG | CCG | EYT2011-12; MD, EYT2012-13; HS, EYT2013-14; MD, EYT2014-15; I, EYT2014-15; MD, EYT2014-15; HS, EYT2015-16; MD | +92-553 |  | MetaQTL-6 | 33 -100 Mb from two stable QTL |
| HB1B.20 | S1B_642616640,  S1B_642616658 | 1B | 18 | AC, AG, GC | GC | EYT2014-15; SD, EYT2016-17; MD, EYT2016-17; HS, EYT2017-18; SD | +79-359 |  | MetaQTL-6 |  |
| HB2B.10 | S2B_24883552,  S2B_24887574,  S2B_24899507,  S2B_24899536,  S2B_24899572 | 2B | 16020 | ACATG, ACGTC, ATGCG,  GCATG | ACATG | EYT2012-13; HS, EYT2016-17; I, EYT2017-18; MD, EYT2017-18; HS | +177-357 |  |  | 81 Mb from stable QTL |
| HB2B.15 | S2B_75832544,  S2B_75848057 | 2B | 15513 | AT, GC | GC | EYT2012-13; DS, EYT2012-13; HS, EYT2015-16; DS, EYT2016-17; HS | +35-265 |  |  | 30 Mb from stable QTL |
| HB2B.42 | S2B_784544719,  S2B_784774250,  S2B_784905791,  S2B_784905811 | 2B | 361092 | AGGC, GACT,  GAGT | AGGC | EYT2011-12; MD, EYT2011-12; HS, EYT2012-13; I, EYT2014-15; SD, EYT2015-16; SD | +123-435 |  |  | 579 Mb from stable QTL |
| HB3B.2 | S3B_7240747,  S3B_7240753 | 3B | 6 | AA, AG, GG | AG | EYT2011-12; MD, EYT2011-12; HS, EYT2012-13; SD, EYT2017-18; HS | +148-449 |  | MetaQTL-27 |  |
| Hap. block | Markers in Hap. block | Chr | Interval; First-Last SNP (bp) | Haplotype alleles | Fav. allele | EYT: Testing environment | Allelic effect | Overlapping GWAS peaks of yield related traits (panels in GrainGenes database) | Overlap with meta-QTL | Overlap with /distance from haplotypes-based stable QTL (Li et al. 2019) |
| HB3B.23 | S3B_758391015,  S3B_758438223,  S3B_758464620,  S3B_758612003,  S3B_758612056,  S3B_758613386 | 3B | 222371 | AAGAGC,  CTCGCT | CTCGCT | EYT2011-12; MD, EYT2011-12; HS, EYT2013-14; MD, EYT2016-17; I, EYT2016-17; SD, EYT2017-18; HS | +27-264 |  |  |  |
| HB4A.20 | S4A_713064971,  S4A_713506269,  S4A_713517340,  S4A_713522176 | 4A | 457205 | CGTC, TACT,  TGTC | CGTC | EYT2011-12; SD, EYT2013-14; I, EYT2013-14; SD, EYT2014-15; HS, EYT2017-18; SD | +85-233 |  |  |  |
| HB4A.25 | S4A_721406670,  S4A_721406696,  S4A_721826603,  S4A_721826636 | 4A | 419966 | GCGA, GTCA,  GTCG,  TCGA | GTCA | EYT2012-13; MD, EYT2013-14; SD, EYT2015-16; I, EYT2015-16; MD | +79-334 |  |  |  |
| HB4A.27 | S4A_730188545,  S4A_730188899 | 4A | 354 | AT, GC | GC | EYT2011-12; SD, EYT2012-13; SD, EYT2012-13; HS, EYT2014-15; I, EYT2014-15; MD | +95-267 |  |  |  |
| HB4B.8 | S4B_644330895,  S4B_644330917 | 4B | 22 | CA, CG, GG | GG, CG | EYT2011-12; I, EYT2012-13; SD, EYT2012-13; HS, EYT2013-14; I, EYT2013-14; MD, EYT2013-14; SD, EYT2016-17; HS | +105-324 |  |  |  |
| HB4B.12 | S4B_663621978,  S4B_663622013 | 4B | 35 | CC, CT, TC | TC | EYT2013-14; I, EYT2014-15; I, EYT2014-15; SD, EYT2016-17; I | +168-429 |  |  |  |
| HB5A.15 | S5A_548234618,  S5A_548234636,  S5A_548387200,  S5A_548422588 | 5A | 187970 | AGAC, ATAG, GGTC | AGAC | EYT2012-13; HS, EYT2014-15; MD, EYT2016-17; I, EYT2017-18; I | +81-297 |  |  | 20 Mb from stable QTL |
| HB5B.3 | S5B_24292046,  S5B_24537970,  S5B_24648800,  S5B_24677091 | 5B | 385045 | AGGG,  CATA | AGGG | EYT2011-12; HS, EYT2012-13; I, EYT2013-14; HS, EYT2017-18; MD | +68-122 | TCAP WUE Hard Winter Wheat AM Panel |  |  |
| HB5B.6 | S5B_47584429,  S5B_47592949 | 5B | 8520 | CT, TC | CT | EYT2011-12; HS, EYT2012-13; SD, EYT2012-13; HS, EYT2013-14; SD, EYT2014-15; SD | +40-249 | Elite HRWAM Panel NUE |  | 45 Mb from stable QTL |
| HB5B.21 | S5B_513712393,  S5B_513713184 | 5B | 791 | AA, GG | AA | EYT2011-12; SD, EYT2012-13; MD, EYT2014-15; I, EYT2015-16; HS | +114-211 |  | MetaQTL-44 | 7 Mb from stable QTL |
| HB5D.5 | S5D_550192169,  S5D_550192174 | 5B | 5 | CC, TA | TA | EYT2012-13; HS, EYT2014-15; I, EYT2016-17; I, EYT2016-17; MD | +116-496 | TCAP WUE Hard Winter Wheat AM Panel |  |  |
| Hap. block | Markers in Hap. block | Chr | Interval; First-Last SNP (bp) | Haplotype alleles | Fav. allele | EYT: Testing environment | Allelic effect | Overlapping GWAS peaks of yield related traits (panels in GrainGenes database) | Overlap with meta-QTL | Overlap with /distance from haplotypes-based stable QTL (Li et al. 2019) |
| HB6B.6 | S6B_17686703,  S6B_17701765 | 6B | 15062 | AG, GC | GC | EYT2011-12; I, EYT2014-15; I, EYT2016-17; I,  EYT2017-18; I | +62-299 |  | MetaQTL-51 |  |
| HB6B.20 | S6B_459374225,  S6B_459374299 | 6B | 74 | CT, TC | CT | EYT2011-12; HS, EYT2012-13; SD, EYT2012-13; HS, EYT2017-18; HS | +53-245 |  |  |  |
| HB6B.38 | S6B_708712113,  S6B_708712131 | 6B | 18 | CG, GA, GG | GA | EYT2011-12; SD, EYT2011-12; HS, EYT2012-13; HS, EYT2014-15; HS | +85-396 |  |  |  |
| HB7A.2 | S7A_7938818,  S7A_7938819 | 7A | 1 | CT, GC, GT | GC | EYT2012-13; HS, EYT2013-14; HS, EYT2014-15; HS, EYT2017-18; SD, EYT2017-18; HS | +96-239 |  |  |  |
| HB7A.3 | S7A_12011058,  S7A_12011069 | 7A | 11 | AT, GA, GT | AT | EYT2011-12; SD, EYT2011-12; HS, EYT2012-13; HS, EYT2013-14; HS, EYT2015-16; HS, EYT2017-18; SD, EYT2017-18; HS | +104-229 | TCAP WUE Hard Winter Wheat AM Panel |  |  |
| HB7B.11 | S7B_124548883,  S7B_124549059 | 7B | 176 | AG, GA | AG | EYT2011-12; SD, EYT2011-12; HS, EYT2012-13; HS, EYT2013-14; SD, EYT2014-15: HS, EYT2017-18; HS | +69-267 |  |  |  |
| HB7B.18 | S7B_576927863,  S7B_576927877 | 7B | 14 | CA, CG, TA | TA | EYT2012-13; HS, EYT2014-15; SD, EYT2015-16; HS, EYT2016-17; MD | +122-470 |  |  |  |
| HB7B.21 | S7B_605313385,  S7B_605313397 | 7B | 12 | AT, GG | AT | EYT2011-12; SD, EYT2012-13; SD, EYT2013-14; HS, EYT2016-17; HS, EYT2017-18; SD | +60-198 |  |  |  |
| HB7B.45 | S7B_733461150,  S7B_733461162 | 7B | 12 | CA, TG | TG | EYT2011-12; SD, EYT2012-13; HS, EYT2013-14; I, EYT2014-15; MD, EYT2016-17; I | +101-189 |  |  |  |
